# Romulus: robust multi-state identification of transcription factor binding sites from DNase-seq data

**DOI:** 10.1093/bioinformatics/btw209

**Published:** 2016-04-19

**Authors:** Aleksander Jankowski, Jerzy Tiuryn, Shyam Prabhakar

**Affiliations:** ^1^Faculty of Mathematics, Informatics and Mechanics, University of Warsaw, 02-097 Warszawa, Poland; ^2^Computational and Systems Biology, Genome Institute of Singapore, Singapore 138672, Singapore

## Abstract

**Motivation:** Computational prediction of transcription factor (TF) binding sites in the genome remains a challenging task. Here, we present Romulus, a novel computational method for identifying individual TF binding sites from genome sequence information and cell-type–specific experimental data, such as DNase-seq. It combines the strengths of previous approaches, and improves robustness by reducing the number of free parameters in the model by an order of magnitude.

**Results:** We show that Romulus significantly outperforms existing methods across three sources of DNase-seq data, by assessing the performance of these tools against ChIP-seq profiles. The difference was particularly significant when applied to binding site prediction for low-information-content motifs. Our method is capable of inferring multiple binding modes for a single TF, which differ in their DNase I cut profile. Finally, using the model learned by Romulus and ChIP-seq data, we introduce Binding in Closed Chromatin (BCC) as a quantitative measure of TF pioneer factor activity. Uniquely, our measure quantifies a defining feature of pioneer factors, namely their ability to bind closed chromatin.

**Availability and Implementation:** Romulus is freely available as an R package at http://github.com/ajank/Romulus.

**Contact:** ajank@mimuw.edu.pl

**Supplementary information:** Supplementary data are available at *Bioinformatics* online.

## 1 Introduction

Eukaryotic transcription is an extremely complex process, controlled by transcription factor (TF) binding to regulatory elements in multiple locations in the genome. The traditional method of analyzing individual active regulatory elements in the genome involves the digestion by DNase I and subsequent identification of regions where TFs are bound to the DNA fragment and protect the DNA from degradation by the enzyme. These protected sites, or TF footprints, can be identified on a large scale by a more recent protocol, DNase I digestion followed by high-throughput sequencing (DNase-seq) (Crawford *et al.*, 2006).

CENTIPEDE (Pique-Regi *et al.*, 2011) was the first algorithm aimed at combining sequence information with chromatin state data to identify the sites where a particular TF was bound in the genome. This method relied on the presence of a DNA sequence motif at candidate binding sites. A logistic regression model allowed for multiple types of prior information to be incorporated, e.g. Position Weight Matrix (PWM) score, distance to the nearest Transcription Start Site and sequence evolutionary conservation. The posterior component consisted of a combination of negative binomial and multinomial positional models for each type of experimental data, such as DNase-seq or histone modification ChIP-seq. Overall, the main strength of CENTIPEDE is the ability to identify binding sites for multiple TFs from a single DNase-seq experiment.

MILLIPEDE (Luo and Hartemink, 2013), a method inspired by CENTIPEDE, also aims at identifying TF binding sites, and also combines DNase digestion data with TF binding specificity information, but performs the analysis in a supervised manner. The supervised approach allows for the model to be explicitly trained to discriminate bound and unbound sites, but requires to provide such a classification as input. The method of MILLIPEDE is a simplification of the CENTIPEDE approach. Instead of a comprehensive combination of negative binomial and multinomial models to represent the positional distribution of DNase I cuts, these cuts were grouped into several (e.g. 5 or 12) bins. The log-transformed DNase I cut counts within these bins were incorporated in the logistic regression model, together with all the prior information. Overall, the number of parameters in MILLIPEDE is at least an order of magnitude smaller than in CENTIPEDE. The authors showed that MILLIPEDE outperforms CENTIPEDE marginally in human but dramatically in yeast. This was attributed mostly to avoiding over-fitting of parameters by focusing on a more coarse-grained set of features, capturing the large-scale differences between the bound and unbound states. The selection of optimal discriminative features is a hallmark of another similar method, BinDNase (Kähärä and Lähdesmäki, 2015), which also follows the supervised approach and outperforms MILLIPEDE.

Wellington (Piper *et al.*, 2013) is another recent algorithm to predict occupied TF binding sites from DNase-seq data. This algorithm is based on a completely different approach, and does not require a DNA sequence motif to identify the prior set of candidate binding sites. Instead, Wellington quantifies an imbalance in the DNA strand-specific alignment information of DNase-seq data around virtually every location in the genome. The authors argue that in a ‘double hit’ DNase-seq experiment, where each end of a DNA fragment represents an *in vivo* DNase I cleavage site, most of the DNA fragments captured for sequencing are in the order of 50–150 bp in length, and they are expected to originate from within the DNase I hypersensitive sites, as opposed to nucleosomal DNA.

Since the length of DNase I hypersensitive sites is usually 200–250 bp, the captured fragments are likely to span the regions of DNA protected by bound TFs. These captured fragments manifest themselves after sequencing as 5′ sequence tags, representing just one end of these fragments. Hence, a typical DNase I hypersensitive site should be enriched in forward strand tags upstream and in reverse strand tags downstream of bound TFs. Wellington takes advantage of this strand imbalance criterion to greatly increase the specificity by reducing the number of false positives. For each basepair, Wellington tests the hypothesis that there are significantly more reads aligning to the forward strand in the upstream shoulder region with respect to the reads aligning to the forward strand in the footprint region. Moreover, a reverse complement hypothesis is tested, i.e. that there are significantly more reads aligning to the reverse strand in the downstream shoulder region with respect to the reads aligning to the reverse strand in the footprint region. The final Wellington *P*-value for a given genomic location is a product of the two for the aforementioned hypotheses.

None of the existing methods mentioned above allows for direct incorporation of knowledge about TF complexes which could be formed by the TF of interest. The comprehensive knowledge of such functional structures is essential to fully understand the mechanisms of transcriptional regulation. Recently, several computational methods are focused on the identification of the putative structures of TF complexes that bind cooperatively to DNA, using ChIP-seq (Whitington *et al.*, 2011) or chromatin accessibility data (Jankowski *et al.*, 2014; Kazemian *et al.*, 2013). Such a prior information on TF complex structures should be incorporated by the methods aimed at identification of individual TF binding sites.

Here, we propose Romulus, a novel computational method to accurately identify TF footprints from genome sequence information and cell-type–specific experimental data, such as DNase-seq data. Our approach combines the strengths of CENTIPEDE and Wellington, while keeping the number of free parameters in the model relatively low. For a given TF, we first identify candidate binding sites that have above-background sequence affinity, using a Position Weight Matrix. Then, following CENTIPEDE, we employ an Expectation–Maximization-based approach to simultaneously learn the DNase I cut profiles and classify the binding sites as bound or unbound by the TF.

Our method allows for multiple bound states for a single TF, differing in their cut profile and overall number of DNase I cuts. To make the model robust, we employ a systematic approach to group the DNase I cuts, according to their location and strand. Inspired by Wellington, we take the forward strand DNase I cuts only upstream and within the binding site, while the reverse strand DNase I cuts only within the binding site and downstream. We model the total number of cuts as a negative binomial component, while the cut distribution (regularized by binning outside the binding site) is modelled as a multinomial component.

Overall, Romulus predictions agree well with experimental ChIP-seq measurements of TF binding at candidate motif instances. We also comprehensively compared the predictive performance of Romulus, CENTIPEDE and Wellington, and show that Romulus significantly outperformed CENTIPEDE and Wellington, especially when applied to DNase-seq datasets with lower sequencing depth. Finally, we introduce Binding in Closed Chromatin (BCC), a single statistic representing the chromatin state component of Romulus for ChIP-seq–inferred binding sites, as a quantitative correlate of pioneer factor activity.

Romulus is available as an R package at http://github.com/ajank/Romulus, along with documentation and examples, under the terms of GNU GPL v3 open source software license.

## 2 Methods

### 2.1 DNase-seq data from multiple sources

The ENCODE Project (Bernstein *et al.*, 2012) provides three different tracks with human DNase-seq data. The track wgEncodeOpenChromDnase from Duke University (Duke) follows the ‘single hit’ protocol (Boyle *et al.*, 2008), while wgEncodeUwDnase and wgEncodeUwDgf, both from University of Washington (UW), follow the ‘double hit’ protocol (Sabo *et al.*, 2006). The latter track, termed Digital Genomic Footprinting (DGF) yields much higher number of sequencing reads (Supplementary Table S1). We used all tracks in three cell types: A549, HepG2 and K562.

### 2.2 ChIP-seq data as a TF binding benchmark

We downloaded a collection of human ChIP-seq datasets (narrowPeak files) from ENCODE and used them as a gold standard for benchmarking TF binding predictions (Supplementary Table S2). This collection of datasets was also used to assess the performance of Wellington in (Piper *et al.*, 2013). The corresponding DNA sequence motifs (Supplementary Table S3) for these TFs were taken from the HOMER (Hypergeometric Optimization of Motif EnRichment) suite (Heinz *et al.*, 2010).

The motif instances in the human genome for these motifs were downloaded from http://homer.salk.edu/homer/ (HOMER Known Motifs track). We considered these motif instances as candidate binding sites (test cases) in performance assessment of the three methods. The reference classification of the candidate binding sites was obtained as follows: motif instances overlapping any ChIP-seq peak were classified as bound, and all the remaining ones were classified as unbound.

### 2.3 Prior probabilities of TF binding

The prior component of the model captures the genomic sequence and other prior (i.e. independent of cell type or conditions) characteristics of the candidate binding site for a given TF. Let us denote by *i* a particular genomic instance (motif match) of a motif of interest. Typically, the prior characteristics assigned to motif instances could be: the respective PWM score, average evolutionary conservation and so on.

To formalize the model, let us denote the value of the *j*th prior characteristic for motif instance *i* by a real number $x_{i}{}^{\left(j\right)}$, where 1 ≤ *j* ≤ *J*. In the simplest case, where each motif instance can be either ‘bound’ or ‘unbound’, we apply a logistic approach to model the ratio of the prior probabilities:

$$P\left(Z_{i}=1\right)/P\left(Z_{i}=0\right)=\exp(\beta_{0}+\sum_{j}\beta_{j}x_{i}{}^{\left(j\right)}).$$

Here, *Z_i_* = 1 indicates that the *i*th motif instance is bound, whereas *Z_i_* = 0 indicates that it remains unbound. Such a model has been used in CENTIPEDE (Pique-Regi *et al.*, 2011).

We generalize the above-described model. Let us consider a TF that manifests one or more cooperative binding modes, with well-defined offsets and orientations within the underlying motif complexes. The cooperative binding modes, and the corresponding motif complexes, will be both denoted by *k *= 2, …, *K *+* *1, where *K* is the number of cooperative binding modes. Each of these complexes imposes certain offset and orientation of the partner motif with respect to the primary motif (Jankowski *et al.*, 2013, 2014). The genomic location of the motif instance *i* therefore implies the corresponding locations for all partner motifs within all defined motif complexes. The prior characteristics for these partner motif instances are calculated no matter how unfavourable they may be for binding, and are included in the sequence *x_i_*^(^*^j^*^)^, where 1 ≤ *j *≤* J*. Note that in the homodimer case, some of these characteristic may be derived from the same PWM, however scored at a different genomic location.

Now let us focus on a particular cooperative binding mode *k*, where 2 ≤ *k *≤* K *+* *1. We introduce binary indicators $\gamma_{\text{j}}{}^{\left(k\right)}\in \left\{0,\text{1}\right\}$, specifying whether the prior characteristic $x_{i}{}^{\left(j\right)}$should be taken into account in a cooperative binding mode *k*. The values of these indicators ensure that only the characteristics specific to the primary motif instance and to the partner motif instances within *k*th motif complex will be taken into account. The monomer binding mode, denoted by *k* = 1, should be characterized only by the characteristics referring to the primary motif instance. Hence, $\gamma_{j}{}^{\left(1\right)}\;=\;0$ for all the characteristics *j* referring to any of the partner motifs. To model the prior probabilities, we apply a logistic model against the unbound ‘pivot’ case of *Z_i_* = 0:

$$P\left(Z_{i}=k\right)/P\left(Z_{i}=0\right)=\exp(\beta_{0}{}^{\left(k\right)}+\sum_{j}\beta_{j}{}^{\left(k\right)}\gamma_{j}{}^{\left(k\right)}x_{i}{}^{\left(j\right)}),$$

where *Z_i_ *= 0 indicates no binding, *Z_i_ *= 1 refers to binding as monomer, and *Z_i_ *= 2, …, *K *+ 1 refer to the respective cooperative binding modes. This way, we have *K *+ 1 outcomes separately regressed against the pivot outcome *Z_i_* = 0 (see Supplementary Methods).

### 2.4 Chromatin state component

The chromatin state component of the model captures the cell-type–specific and condition-specific experimental data around the candidate binding sites. Typically, these experimental data will include DNase-seq or histone modification ChIP-seq profiles. In this study, we use DNase-seq profiles around the candidate binding sites, but other types of data might be used as well.

For each motif instance *i*, we consider the numbers of DNase I cuts at individual basepairs in the vicinity in a strand-specific manner. The forward strand cuts are taken only upstream and within the candidate binding site, while the reverse strand cuts only within the candidate binding site and downstream. In this study, we consider a 200 bp margin. Assuming that *L* is the length of the motif, the matrix DNase^+^*_i_*_,_*_j_* contains the numbers of forward strand DNase I cuts (genomic positions *j* = 1, …, 200 + *L*, relative to the position of motif instance, starting 200 bp upstream of the motif, continuing downstream), and the matrix DNase^−^*_i_*_,_*_j_* contains the numbers of reverse strand cuts (*j* = *1*, …, 200 + *L*, starting at the motif, continuing downstream). We consider such matrices for all the chromatin state data available to the model.

We also observed that all kinds of datasets based on short sequence reads, in particular all kinds of DNase-seq data, are prone to artifactual spikes of reads (above 100 reads) mapped to a single location and strand in the genome. These spikes may arise from the sequence fragments originating at repetitive regions with incomplete representation in the reference genome. Hence, we applied clipping to the number of reads mapped to a single location and strand, choosing the threshold as the value of 99.9% quantile of all the entries (for all *i* and *j*) of both matrices. The values above the threshold were set to be equal to the threshold itself. We tried to use other quantiles apart from the 99.9% quantile, namely 99% and 99.99%; they all gave similar results (data not shown).

Let $X_{i}\;=\;({\left(\text{DNase}{\;}^{+}{\;}_{i}{}_{,j}\right)}_{j},{\left(\text{DNase}{\;}^{-}{\;}_{i}{}_{,j}\right)}_{j},\ldots )$ denote all the chromatin state data available to the model for a given motif instance *i*. As stated in the previous subsection, we introduce latent variables *Z_i_* such that $P\left(Z_{i}\;=\;0|X_{i}\right)$ is the probability of motif instance *i* to be unbound, $P\left(Z_{i}\;=\text{ 1 }|X_{i}\right)$ is the probability of it being bound by monomer, $P\left(Z_{i}\;=\text{ 2 }|X_{i}\right)$ is the probability of it being bound in the first cooperative binding mode, and so on. Our primary interest is

$$p_{i}=\sum_{k=\text{1}}^{K+\text{1}}P(Z_{i}=k|X_{i})=1-P(Z_{i}=0|X_{i}),$$

i.e. the probability of the motif instance *i* to be bound in any binding mode. It follows from the Bayes' theorem (see Supplementary Methods) that

$$\frac{p_{i}}{\text{1}-p_{i}}=\sum_{k=\text{1}}^{K+\text{1}}\frac{P\left(X_{i}|Z_{i}=k\right)P\left(Z_{i}=k\right)}{P\left(X_{i}|Z_{i}=\text{0}\right)P\left(Z_{i}=\text{0}\right)},$$

where $P\left(Z_{i\;}{=}_{\;}k\right)/P\left(Z_{i}\;=\;0\right)$ is the prior component of the model discussed in the previous subsection.

The remaining conditional probabilities $P\left(X_{i}|Z_{i\;}{=}_{\;}k\right)$ for *k* = 0, …, *K *+ 1 are modelled as follows. We assume that all the chromatin state data included in the model are independent, given its binding state *Z_i_*. Hence, the conditional probability is a product of the corresponding conditional probabilities for each type of chromatin state data:

$$\text{P}\left(X_{i}|Z_{i}=k\right)=\text{P}\left({\left({\text{DNase}}^{+}{{}_{i}}_{,j}\right)}_{j}|Z_{i}=k\right)\cdot \text{P}\left({\left({\text{DNase}}^{-}{{}_{i}}_{,j}\right)}_{j}|Z_{i}=k\right)\cdot \ldots .$$

Each of the conditional probabilities on the right side is modelled separately, using a factorized model. For brevity, we discuss the parametrization only for $P\left({\left(\text{DNase}{\;}^{+}{\;}_{i}{}_{,j}\right)}_{j}|Z_{i\;}{=}_{\;}k\right)$, i.e. forward strand DNase I cuts; it is analogous for the other strand and for other types of data. The first, negative binomial component captures the total number of reads mapped in the vicinity of the motif instance *i* in binding mode *k.* The negative binomial distribution is naturally parametrized by the success probability $p^{+\left(k\right)}\in \left(0,\text{1}\right)$ and the real-valued number of failures $r^{+\left(k\right)}\;>\;0$. The second, multinomial component quantifies the probability of a particular spatial distribution of the given total number of DNase I cuts, using the multinomial distribution (see Supplementary Methods for details).

As opposed to CENTIPEDE (Pique-Regi *et al.*, 2011), we do not keep a separate free parameter for each position (respective to the motif location) and strand, but apply a more flexible approach. For each binding mode *k*, we divide the positions *j* into one or more bins. Let us denote by DNaseBin*_j_*^+(^*^k^*^)^ the bin number for position *j* in binding mode *k*. Note that binding modes may differ in the way the positions are split into bins. In this study, we take 20 bp long bins outside the motif site, and single-basepair bins within the motif site. Moreover, for the unbound mode (*k* = 0) we put all the positions in a single bin.

For a given binding mode *k*, we associate a free parameter $\lambda_{b}{}^{+\left(k\right)}$ with each bin *b* = 1, …, *B*^+(^*^k^*^)^. However, for the multinomial distribution we must provide a vector of probabilities covering every single position in the vicinity of the motif instance. Hence, we calculate the actual multinomial coefficients ${ƛ}_{j}{{}^{+}}^{\left(k\right)}$ by taking the values of $\lambda_{b}{}^{+\left(k\right)}$ for *b *=* *DNaseBin*_j_*^+(^*^k^*^)^ and normalizing them so that $\sum_{j}{ƛ}_{j}{{}^{+}}^{(}{{}^{k}}^{)}\;=\text{ 1}$. By definition, the multinomial coefficients ${ƛ}_{j}{{}^{+}}^{\left(0\right)}$for the unbound state are equal, i.e. there is no positional preference for DNase I cuts in the null model.

### 2.5 Expectation–Maximization approach

To estimate the model parameters 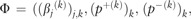

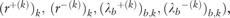
 we apply the Expectation–Maximization approach using a complete likelihood function. We found no closed-form solution for ${{{(\beta }_{j}}^{\left(k\right)})}_{j,k},{\left(r^{+(k)}\right)}_{k}$ and ${\left(r^{–(k)}\right)}_{k}$ that maximizes the likelihood function, hence we apply the Broyden–Fletcher–Goldfarb–Shanno (BFGS) numerical optimization procedure (see Supplementary Methods). To increase the robustness of the model, we employ a shrinkage estimator of the parameters ${\left(\lambda_{b}{}^{+(k)}\right)}_{b,k}$ and ${\left(\lambda_{b}{{}^{-}}^{\left(k\right)}\right)}_{b,k}$. For each *b* and *k*, we take the regularized estimator $\delta \lambda_{b}{}^{+(k)}\;+\;(\text{1 }-\;\delta )\left|J_{b}\right|/\sum_{b}\left|J_{b}\right|$, where $\left|J_{b}\right|$ is the size of the bin *b*, and δ is the mixing parameter, equal to 0.5 by default.

The Expectation–Maximization procedure was initialized by assigning the prior probabilities as follows. In the monomer binding mode, we put $P\left(Z_{i}\;=\text{ 1}\right)/P\left(Z_{i}\;=\;0\right)\;=\text{ 1}00$ for the top 10% of motif instances with highest total number of DNase-seq cuts. In a dimer binding mode *k*, we put $P\left(Z_{i\;}{=}_{\;}k\right)/P\left(Z_{i}\;=\;0\right)\;=\text{ 1}00$ for the motif instances satisfying both of the following criteria: being within the top 10% of motif instances with highest total number of DNase-seq cuts, and being within the top 10% of motif instances with highest dimerization partner motif score. In the cases not mentioned above for any bound mode *k*, we put $P\left(Z_{i\;}{=}_{\;}k\right)/P\left(Z_{i}\;=\;0\right)\;=\;0.0\text{1}$. This default initialization procedure allows for robust convergence of the algorithm; other procedures may perform similarly (Supplementary Fig. S9; see Supplementary Methods).

We then estimate the values for ${{{(\beta }_{j}}^{\left(k\right)})}_{j,k}$, and for the first Maximization step we take the prior probabilities as the posterior ones. We iterate the Expectation–Maximization procedure, in each iteration getting a revised vector of parameters, until the posterior probabilities do not change by more than 0.001. In most of the cases described here, the algorithm converged in fewer than 30 iterations.

## 3 Results

### 3.1 Romulus systematically outperforms existing methods

We systematically benchmarked Romulus along with two abovementioned tools for TF binding site identification, CENTIPEDE (Pique-Regi *et al.*, 2011) and Wellington (Piper *et al.*, 2013). As described in the previous section, we applied all the methods in an unsupervised manner to DNase-seq data from three ENCODE sources: Duke DNase, University of Washington (UW) DNase and UW Digital Genomic Footprinting (DGF). From each of the DNase-seq data sources, sequence tag profiles were fetched for three human cell types: A549 (lung adenocarcinoma epithelial cell line), HepG2 (hepatocellular carcinoma cell line) and K562 (leukemia cell line). To validate the predictions, we used 39 ChIP-seq datasets from ENCODE to define genuine TF binding sites (see Methods); note that no ChIP-seq data were used for training.

**Fig. 1.**
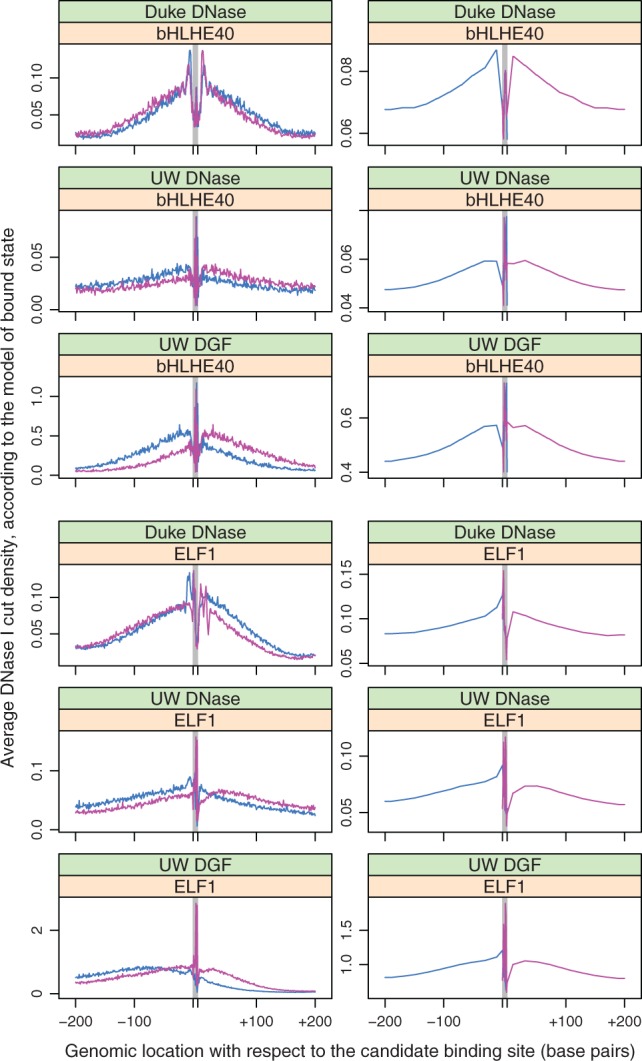
Example models of TF footprints learned by CENTIPEDE (left) and Romulus (right) for two TFs: bHLHE40 (top) and ELF1 (bottom) in A549 cell line. Line colours indicate the strandness of DNase I cuts. In the case of Romulus, the forward strand cuts are considered only upstream and within the binding site, while the reverse strand cuts are considered only within the binding site and downstream (Color version of this figure is available at *Bioinformatics* online.)

Since both Romulus and CENTIPEDE learn a model for TF footprints, we visualized these models by plotting their multinomial components for representative cases (Fig. 1). Clearly, the DNase I cut profiles fitted by CENTIPEDE capture much more noise than their Romulus counterparts. Note that the curves for Romulus were smoothed for the purpose of visualization by replacing the fixed-value bins by a piecewise linear function. The multinomial models for Romulus were based on a far smaller number of free parameters than their CENTIPEDE counterparts (e.g. 48 versus 825 free parameters for 10 bp motif and 200 bp margin), hence they are less prone to overfitting.

To systematically benchmark the three tools, we assessed their predictive power for each combination of cell type and TF for which we have reference ChIP-seq data (see Methods). The standard approach in assessing binary classifier performance is the Receiver Operating Characteristic (ROC) curve, showing the relationship between false positive rate (i.e. 1 − specificity, *x* axis) and true positive rate (i.e. sensitivity, *y* axis). However, albeit routinely performed, application of ROC curves to assess the performance of TF binding site prediction is not adequate. These classification tasks are characterized by highly skewed datasets containing relatively small number of true positives (motif instances actually bound by the TF) and large number of true negatives (motif instances that remain unbound). Hence, the shape of ROC curves and area under them is mostly affected by the ability of a particular tool to correctly predict unbound motif instances (Piper *et al.*, 2013).

**Fig. 2.**
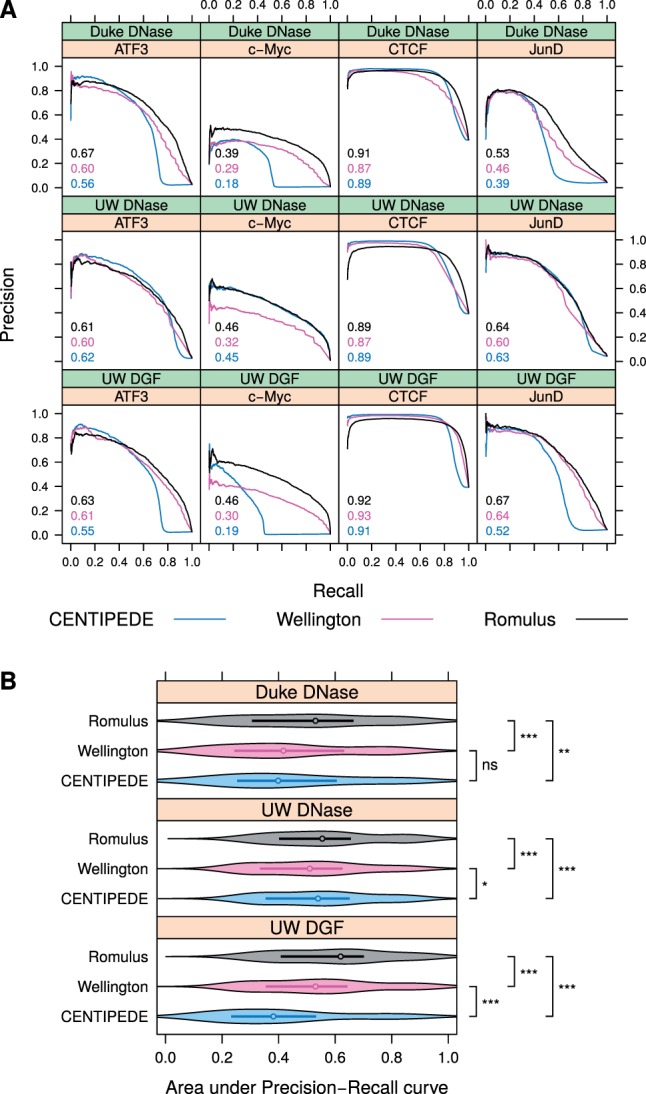
Prediction performance of CENTIPEDE, Wellington and Romulus. (**A**) Example Precision-Recall curves in K562 cells using three sources of DNase-seq data. Areas under these Precision-Recall curves are indicated. Only the results for a subset of four representative TFs are shown. (**B**) Areas under Precision-Recall curves aggregated as violin plots and compared between three tools and three DNase-seq data sources. Median values and interquartile ranges are indicated. All the TFs and cell lines (A549, HepG2 and K562) were considered jointly in this panel. ***, *P*-value < 0.001. **, *P*-value < 0.01. *, *P*-value < 0.05. ns, non-significant (Color version of this figure is available at *Bioinformatics* online.)

To address the above-mentioned problem, we plotted Precision-Recall curves, showing the relationship between recall (i.e. sensitivity, *x* axis) and precision (*y* axis). These curves were found to give a more informative picture of an algorithm’s performance when classifying highly skewed datasets (Davis and Goadrich, 2006). Example Precision-Recall curves for several TFs in K562 cells (Fig. 2A) suggest that Romulus outperforms the existing methods in most of the cases. This observation is also supported by the corresponding ROC curves (Supplementary Fig. 1A).

Common single-dimensional measures in assessing classifier performance are the area under ROC curve (AUC-ROC) and the area under Precision-Recall curve (AUC-PR). We calculated these statistics for all the combinations of cell types and TFs considered, for each of the three DNase-seq data sources (Supplementary Figs S2 and S3), and found that indeed Romulus outperformed the other tools in most of the cases. A similar trend was observed for Spearman correlation of binding predictions with ChIP-seq peak height (Supplementary Fig. S4).

To obtain a summary view of the performance of each of the three tools considered (CENTIPEDE, Wellington and Romulus) we aggregated the AUC-PR statistics as violin plots, combining them across different cell types and TFs (Fig. 2B). We used two-sided Wilcoxon signed-rank test to compare the statistics between three tools for each DNase-seq data source, and found that Romulus systematically and significantly outperformed the other tools. The same trend was observed using AUC-ROC statistics (Supplementary Fig. S1B). These aggregations also suggests that the performance of CENTIPEDE is most affected by the choice of DNase-seq data source, while the performance of Romulus and Wellington remain stable. Intriguingly, CENTIPEDE performed poorly for the datasets from UW DGF, despite the fact that UW DGF datasets had at least 2.25 times more reads than the corresponding Duke and UW DNase datasets (Supplementary Table S1).

### 3.2 Romulus improves TF binding site prediction for low-information-content motifs

**Fig. 3.**
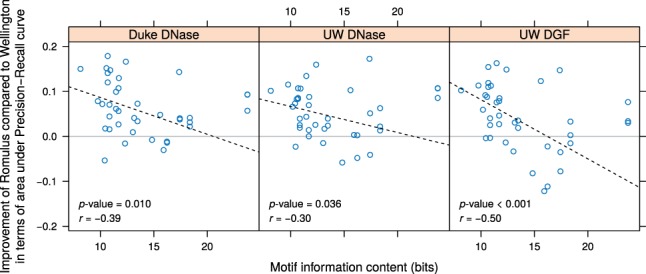
Improvement of Romulus compared to Wellington in terms of area under Precision-Recall curve significantly correlates with motif information content. All the cell lines (A549, HepG2 and K562) were considered jointly here. Pearson correlation values and *P*-values were calculated after excluding the outliers with information content above 20 bits

We also studied the difference between AUC-PR performance of Romulus and the second best performer, Wellington, and found that for some specific TFs the difference is noticeably higher. We hypothesized that this may be related to the motif information content of these TFs. To test this hypothesis, we calculated the Pearson correlation coefficient *r* of the difference between AUC-PR performance, as described above, and the motif information content (Fig. 3). A similar calculation was performed for AUC-ROC (Supplementary Fig. S5).

We also performed a 1000-fold permutation test to calculate the empirical *P*-value for the Pearson correlation coefficient, randomly pairing the AUC-PR performance and the motif information content. For all the three DNase-seq data sources, we observed a statistically significant negative correlation: *P *=* *0.005 for Duke, *P *=* *0.024 for UW DNase-seq data and *P *<* *0.001 for UW DGF (Fig. 3). Thus, the gain of predictive power of Romulus over Wellington is significantly higher for TFs with low-information-content motifs, perhaps because of the Romulus' ability to capture the shape of the DNase I cut profile.

### 3.3 Knowledge of TF dimerization modes does not improve the prediction of individual TF binding sites

Next, we tested the hypothesis that accounting for dimer binding modes could significantly improve TF-DNA binding predictions. The TFs AR, FOXA1, SOX2 and OCT4 are known to form strongly cooperative dimeric binding complexes (Jankowski *et al.*, 2013). We therefore used matching ChIP-seq datasets (Supplementary Table S4) to benchmark the binding predictions made by Romulus for these TFs when information on their dimer binding modes was supplied. We considered putative partner binding sites positioned at a specific offset and orientation with respect to the primary motif, and included the motif scores at these positions as prior characteristics in the prior component of the model (see Methods).

Apart from the known AR–AR homodimer (Nelson *et al.*, 1999) and AR–FOXA1 heterodimer (Wang *et al.*, 2011), we included two predicted FOXA1–FOXA1 homodimers: one with convergent, and one with divergent motifs (Jankowski *et al.*, 2013; Starick *et al.*, 2015). Hence, the Romulus model had four states for AR (i.e. AR monomer, AR–AR, AR–FOXA1 and unbound), and five states for FOXA1 (FOXA1 monomer, FOXA1–AR, FOXA1–FOXA1 divergent, FOXA1–FOXA1 convergent, unbound). The performance of TF binding site prediction was assessed in unstimulated, as well as androgen-stimulated, LNCaP cells.

For SOX2 and OCT4, we included the canonical SOX2–OCT4 heterodimer (Ng *et al.*, 2012). The Romulus model had three states for SOX2 (i.e. unbound, SOX2 monomer and SOX2–OCT4 heterodimer), and three states for OCT4 (i.e. unbound, OCT4 monomer and OCT4–SOX2). The performance of TF binding site prediction was assessed in H1-hESC embryonic stem cells.

**Fig. 4.**
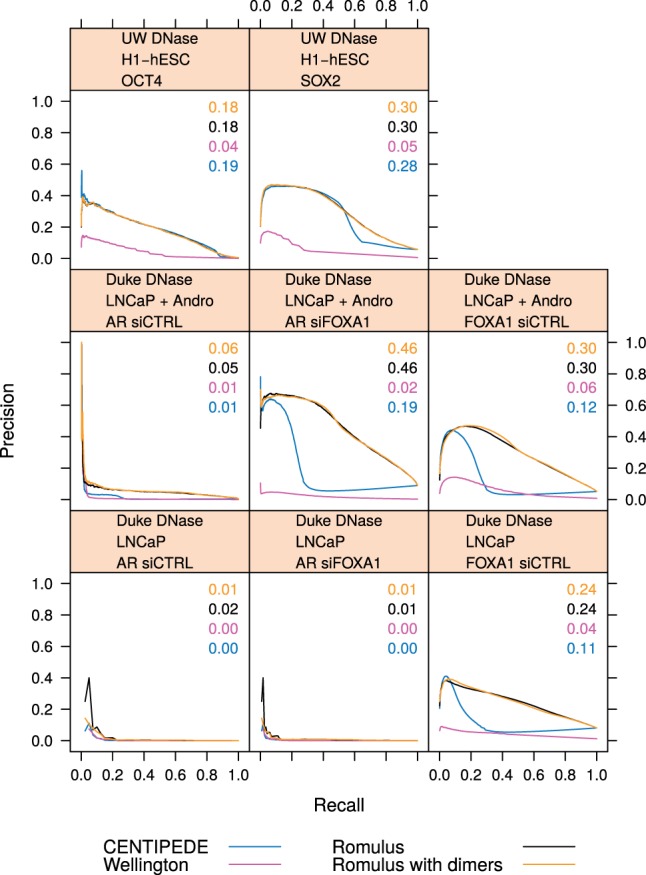
Precision-Recall curves for the known dimers. The TF in focus (OCT4, SOX2, AR or FOXA1), DNase-seq data source (UW or Duke) and conditions are indicated. + Andro, androgen stimulated cells. siFOXA1, silenced FOXA1. siCTRL, control (Color version of this figure is available at *Bioinformatics* online.)

We expected that the additional binding modes would improve overall predictive power, given that prior information on partner motif scores could facilitate separation of distinct dimer footprints. However, in terms of Precision-Recall curves (Fig. 4) and ROC curves (Supplementary Fig. S6) we found no observable improvement. We also confirmed that predicted dimer binding amounted for 32–71% of total predicted binding for a given TF, DNase-seq data source and condition. To verify whether the dimer binding modes indeed have distinguishable profiles, we plotted the components of the Romulus model: the negative binomial component (Supplementary Fig. S7) and the multinomial component (Supplementary Fig. S8). We found that the models learned by Romulus clearly differ between the binding modes, yet their inclusion does not improve the prediction of individual TF binding sites.

### 3.4 Binding in Closed Chromatin as a quantitative predictor of pioneer factor activity

**Fig. 5.**
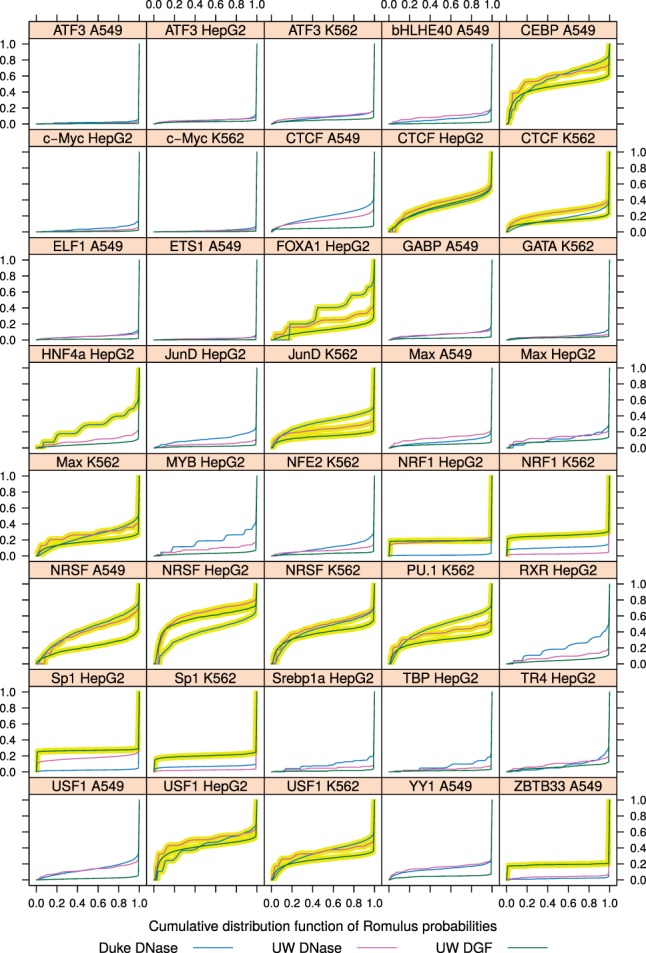
Cumulative distribution functions of the chromatin state component. For each combination of TF and cell type, the cumulative distribution functions of the chromatin state component calculated by Romulus using three sources of DNase-seq data are shown. Cumulative distributions corresponding to BCC values above the threshold from Figure 6 (for each cell type, one MAD above the median) are highlighted (Color version of this figure is available at *Bioinformatics* online.)

We noted that in the case of several TFs it was particularly challenging to predict their binding, and we hypothesized that it might be because these TFs are able to bind closed chromatin in violation of the assumptions of Romulus and other algorithms. In such a situation of binding to nucleosomal DNA, the way Romulus model accounts for the local chromatin openness profile is not necessarily appropriate. We found it promising to quantify this discrepancy. To this end, we limited the scope to the bound motif instances according to the ChIP-seq data, and considered the probabilities of the chromatin state component in the Romulus model. We then plotted the cumulative distribution functions of these probabilities (Fig. 5). These functions should be perceived having two model cases in mind. If a particular TF can only bind open chromatin, its binding sites will all have high chromatin state component probabilities. The cumulative distribution function will therefore remain flat early on and then show a spike towards the right end of the x-axis.

In contrast, a TF that can also bind closed chromatin will tolerate lower chromatin component scores. Its cumulative distribution function will show some elevation at low-to-moderate scores, i.e. in the middle of the x-axis range. Such an ability to bind closed chromatin without the aid of other TFs is a defining feature of pioneer factors. Several pioneer factors have been identified so far, including the forkhead box A (FOXA) factors, GATA-binding (GATA) factors, PU.1 and FOXD3 (Zaret and Carroll, 2011).

To quantify the amount of TF binding that takes place in loci without a pronounced local chromatin openness signal, we introduce Binding in Closed Chromatin (BCC). The value of BCC is calculated as the Area-Under-Curve of a cumulative distribution function described above (Fig. 5). Note that we take only the chromatin state component, and exclude the prior (genomic sequence) component. Incorporation of the primary motif score would be unreliable here, since for some TFs their binding to closed chromatin may be achieved only by their half-site, as is the case for Pax7 (Budry *et al.*, 2012). We observed that the chromatin state component probabilities showed no correlation with ChIP-seq peak height or rank (data not shown). We also compared the BCC values with Spearman correlation coefficient between DNase I accessibility and motif score (Blatti *et al.*, 2015), and found no evident relationship between them (Supplementary Fig. S10, see Supplementary Methods).

**Fig. 6.**
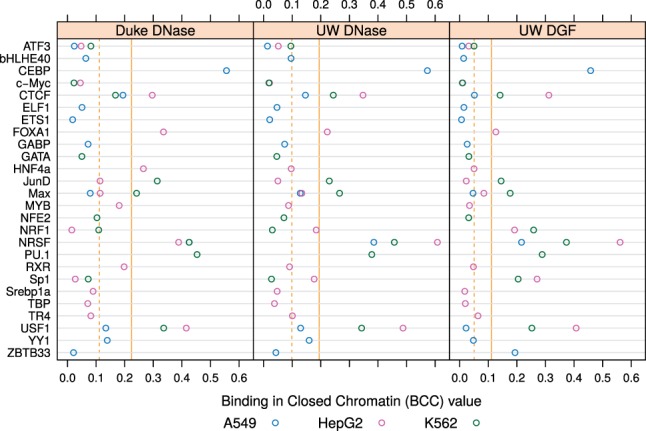
Binding in Closed Chromatin (BCC) values. The value of BCC is shown for each combination of TF, DNase-seq data source and cell type. For each DNase-seq data source, dashed vertical line indicates the mean and solid vertical line indicates the threshold of one MAD (median absolute deviation) above the median (Color version of this figure is available at *Bioinformatics* online.)

We found that the median value of BCC, for all TF binding site predictions in all cell types, was 0.111 in Duke DNase, 0.099 in UW DNase and 0.051 in UW DGF (Fig. 6). We focused on the TFs that had a BCC value, in at least one case, more than one MAD (median absolute deviation) above the median (Fig. 5, highlighted cumulative distribution functions). Eight out of 26 TFs had BCC values above the threshold in at least three cases; these candidate pioneer factors are: CEBP, CTCF, FOXA1, JunD, Max, NRSF, PU.1 and USF1.

Notably, FOXA1 is a canonical example of a pioneer factor (Lupien *et al.*, 2008). We also noted that CTCF and NRSF both have have long, information-rich motifs, which may allow them to bind with high specificity regardless of local chromatin openness. It has been also suggested (Garber et al., 2012) that at an early stage in lineage commitment of dendritic cells, PU.1 and CEBP act as master pioneer factors, while binding of second-tier factors JunB, IRF4 and ATF3 primes the response and sets the basal expression levels of other genes. Our results also suggest a previously unreported pioneer factor activity of upstream stimulatory factor 1 (USF1) in HepG2 and K562 cell lines.

## 4 Discussion

In this study, we comprehensively compared the predictive performance of three tools aimed at predicting TF binding sites from DNase-seq data. Two of them, CENTIPEDE and Wellington, used completely different approaches to address this problem. In our method proposed here, Romulus, we combined the benefits of both of the two other tools, and showed that Romulus significantly outperformed CENTIPEDE and Wellington, regardless of the DNase-seq protocol used (‘single hit’ or ‘double hit’). The advantage of Romulus was observed especially when applied to binding site prediction for low-information-content motifs. It is likely that Romulus gained in robustness and predictive power by limiting the number of free parameters in the multinomial component to 2 · (motif width + 9). In contrast, CENTIPEDE used 2 · (motif width + 400) − 1 free parameters in the multinomial component, and Wellington does not estimate any model parameters from the data.

When allowing for more than one bound state in Romulus, we found that the additional DNase I cut profiles can differ greatly. However, the inclusion of these additional states for the known TF dimers did not yield an increase in predictive power. We hypothesize that three factors potentially contributed to this effect. Firstly, dimer binding sites constitute only a small fraction of all binding sites, and thus the overall improvement from considering dimer binding modes is necessarily limited. Secondly, dimer binding may not induce strong changes in the DNase I cut profile, relative to monomer binding. Rather, the profile shape could be influenced to a greater degree by other parameters, such as the width of the hypersensitive region, the location of the binding site in question with respect to the centre of the region, and the spatial distribution of neighbouring binding sites. If so, the shape of the DNase I cut profile would be only weakly indicative of the binding mode. Finally, inclusion of additional binding modes substantially increases the number of free parameters in the model, which increases the likelihood of overfitting.

A recent computational method, protein interaction quantitation (PIQ), used the correlation between TF binding and chromatin openness to identify TFs that function as pioneer factors (Sherwood *et al.*, 2014). We have shown the value of a different approach that prioritizes binding sites in closed chromatin. We propose that this Binding in Closed Chromatin (BCC) score is a quantitative correlate of pioneer factor activity. In other words, BCC values constitute a measure of a TF's ability to bind nucleosome-occluded DNA. Indeed, multiple known pioneer factors were detected as having high BCC scores. Notably, while PIQ requires data from two different experimental conditions (before and after differentiation, for example) to identify pioneer factors, the calculation of BCC requires data from one condition only. Note that the BCC scores of some TFs varied significantly across cell types. While some of this variability could be attributable to biological factors such as variation in TF expression, technical factors may also play a role. For example, closed-chromatin binding sites, which tend to be weaker, would be detected in smaller numbers in the noisier ChIP-seq datasets.

Intriguingly, CTCF and NRSF (REST) were predicted both by BCC and PIQ as pioneer factors, despite the use of divergent strategies by the two algorithms. A unique feature shared by these two TFs is that they have long, information-rich PWMs (both 20 bp; 32.7 and 34.9 bits of information, respectively) and bind DNA strongly using multiple zinc-finger domains. They could therefore potentially bind DNA ‘alone,’ without requiring co-binding by neighbouring factors over 50–100 bp. In such a scenario, CTCF and NRSF could tightly bind a 20-bp stretch of DNA closely flanked by nucleosomes, leaving little room for DNA cleavage by DNase I. This would explain the high BCC scores of these two TFs. On the other hand, given their high DNA affinity, they would also be likely to bind motif instances in promoter or enhancer regions characterized by highly open chromatin. Thus, they would be predicted as pioneer TFs by both methods, but for different reasons. Pioneer TFs with fewer DNA-binding domains could adopt yet another configuration: they have been shown to bind DNA that is wrapped around a histone octamer (Iwafuchi-Doi and Zaret, 2014), Given the diversity of such possibilities, we anticipate that TF-DNA binding predictions will eventually need to account for multiple binding ‘strategies,’ each defined by the precise nature of the interplay between the TF and nucleosomes.

.

## Funding

This work was supported by the National Science Centre, Poland [DEC-2012/05/B/NZ2/00567 to J.T.]; and the Agency for Science, Technology and Research (A*STAR), Singapore.

*Conflict of Interest*: none declared.

## Supplementary Material

Supplementary Data
