# Supplementary material for: Romulus: robust multi-state identification of transcription factor binding sites from DNase-seq data
Source: Bioinformatics. 2016 Apr 19;32(16):2419–26. doi: 10.1093/bioinformatics/btw209 (PMC4978937; doi:10.1093/bioinformatics/btw209)
Supplement: Supplementary Data [file supp_32_16_2419__index.html]

Romulus: Robust multi-state identification of transcription factor binding sites from DNase-seq data — Romulus: robust multi-state identification of transcription factor binding sites from DNase-seq data — Romulus: robust multi-state identification of transcription factor binding sites from DNase-seq data — Supplementary Data 

# Romulus: robust multi-state identification of transcription factor binding sites from DNase-seq data

## Supplementary Data

files

- Supplementary Data - pdf file
