# Supplementary material for: Romulus: robust multi-state identification of transcription factor binding sites from DNase-seq data
Source: Bioinformatics. 2016 Apr 19;32(16):2419–26. doi: 10.1093/bioinformatics/btw209 (PMC4978937; doi:10.1093/bioinformatics/btw209)
Supplement: Supplementary Data [file supp_btw209_SupplementaryData.pdf]

Romulus: Robust multi-state identification of transcription factor binding sites from DNase-seq data

Supplementary Figures, Tables and Methods

Aleksander Jankowski Jerzy Tiuryn Shyam Prabhakar

March 23, 2016

Supplementary Figures

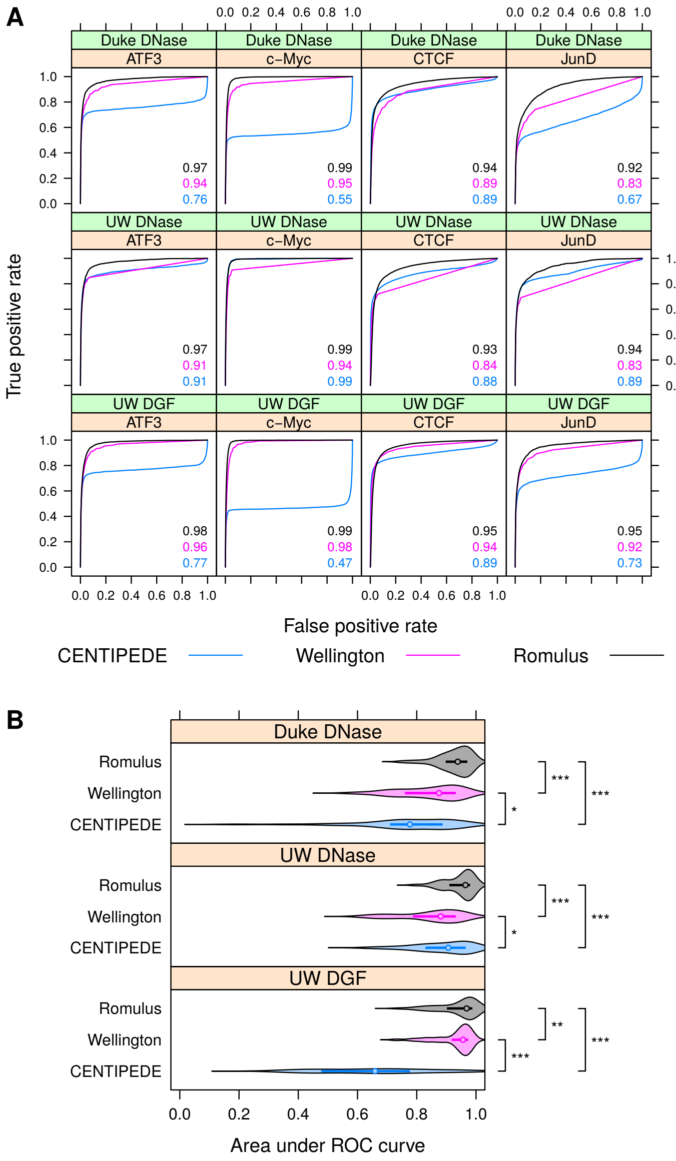

Supplementary Figure 1. Prediction performance of CENTIPEDE, Wellington and Romulus. (A) Example Receiver Operating Characteristic curves in K562 cells using three sources of DNase-seq data. Areas under these ROC curves are indicated. Only the results for a subset of 4 representative TFs are shown. (B) Areas under ROC curves aggregated as violin plots and compared between three tools and three DNase-seq data sources. Median values and interquartile ranges are indicated. All the TFs and cell lines (A549, HepG2 and K562) were considered jointly in this panel. ***, p-value < 0.001. **, p-value < 0.01. *, p-value < 0.05. ns, non-significant.

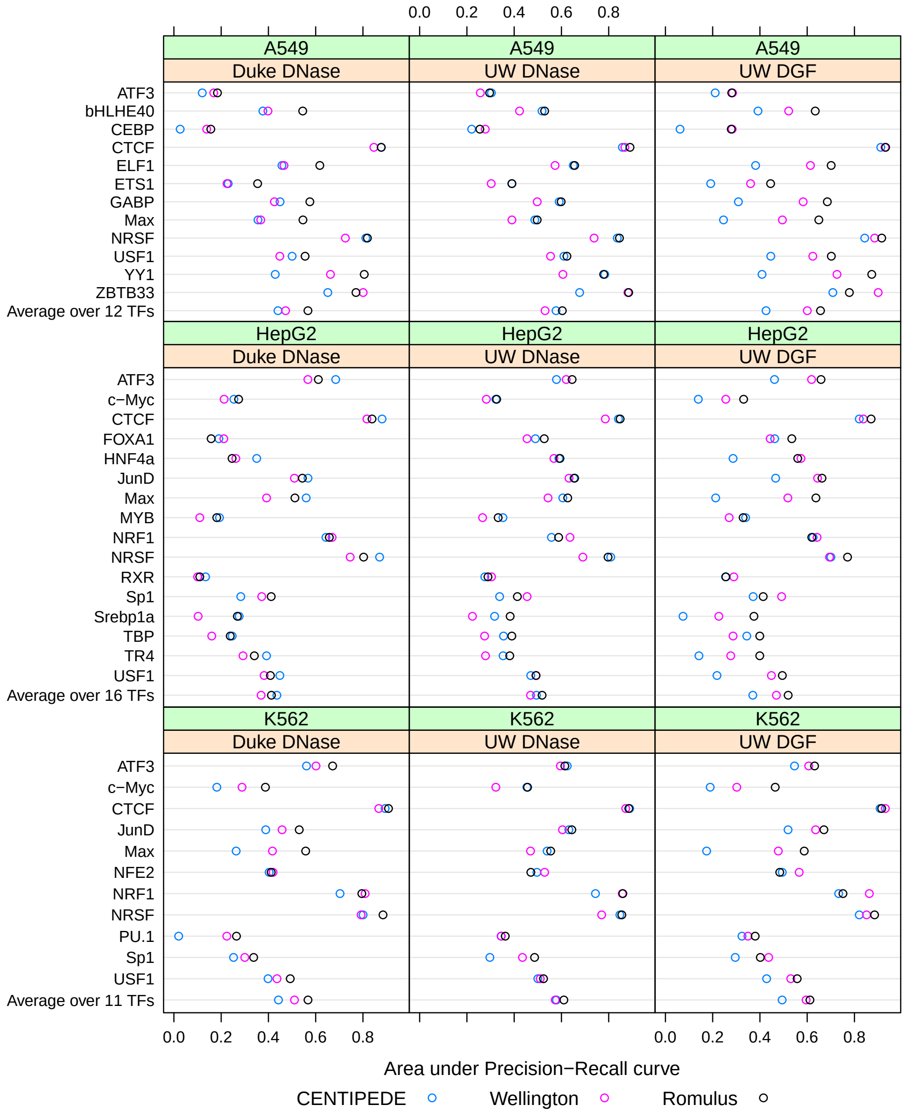

Supplementary Figure 2. Romulus outperforms the other tools in terms of area under Precision-Recall curve. Prediction performance of CENTIPEDE, Wellington and Romulus in A549, HepG2 and K562 cells using three sources of DNase-seq data is shown. Apart from the AUC-PR values for individual TFs, the averages are indicated for each cell line.

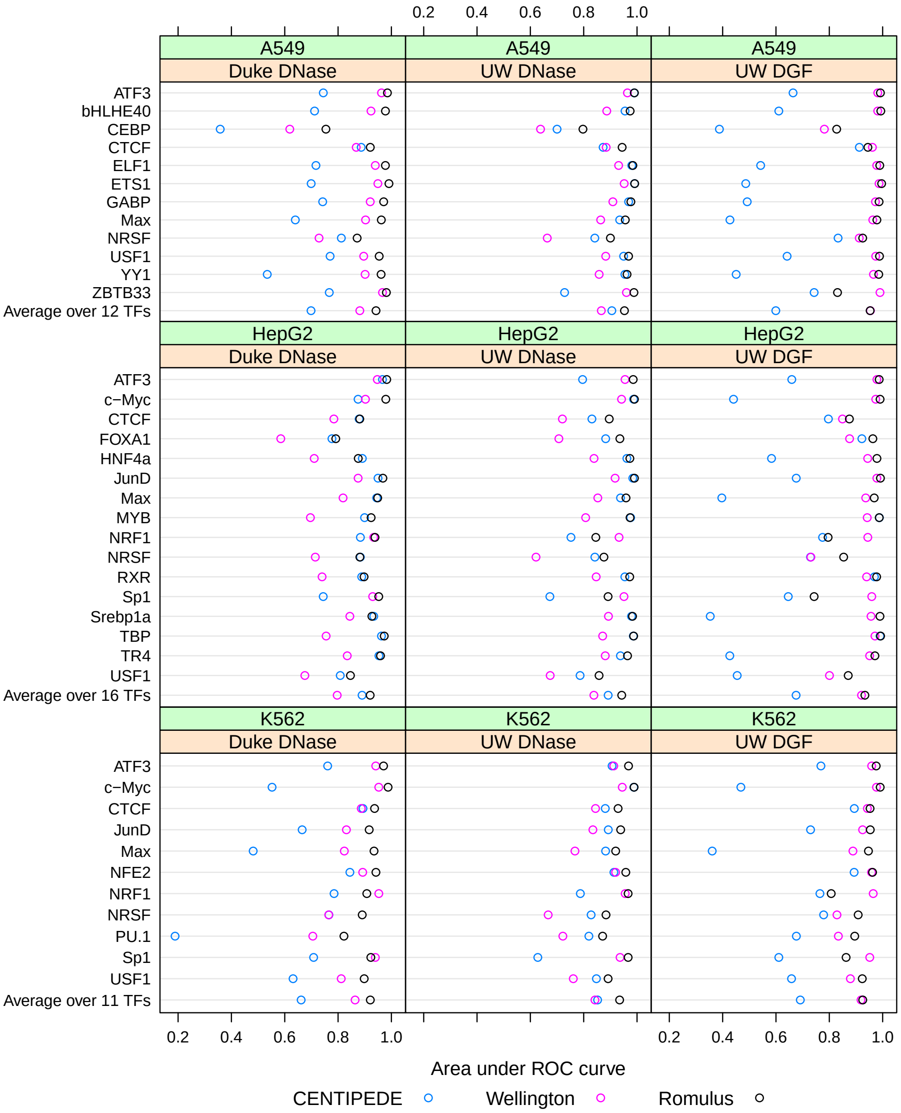

Supplementary Figure 3. Romulus outperforms the other tools in terms of area under Receiver Operating Characteristic curve. Prediction performance of CENTIPEDE, Wellington and Romulus in A549, HepG2 and K562 cells using three sources of DNase-seq data is shown. Apart from the AUC-ROC values for individual TFs, the averages are indicated for each cell line.

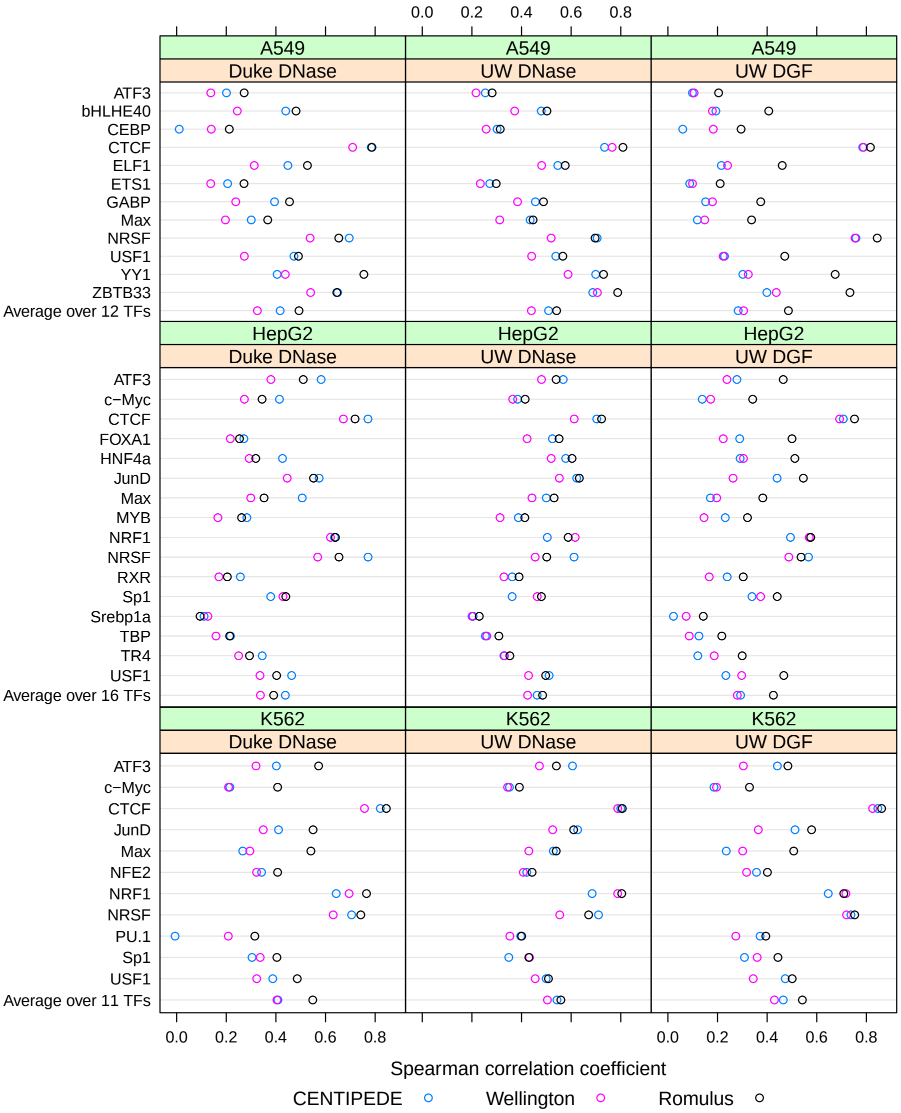

Supplementary Figure 4. Romulus outperforms the other tools in terms of Spearman correlation coefficient between binding predictions and ChIP-seq peak height. Prediction performance of CENTIPEDE, Wellington and Romulus in A549, HepG2 and K562 cells using three sources of DNase-seq data was assessed by applying each tool to calculate the binding probability (CENTIPEDE and Romulus: posterior probability, Wellington: $1-(p\text{-value})$). The probabilites less than 0.5 were clamped down to 0, and the Spearman correlation coefficients between these probabilites and ChIP-seq peak height were calculated. Apart from the correlation values for individual TFs, the averages are indicated for each cell line.

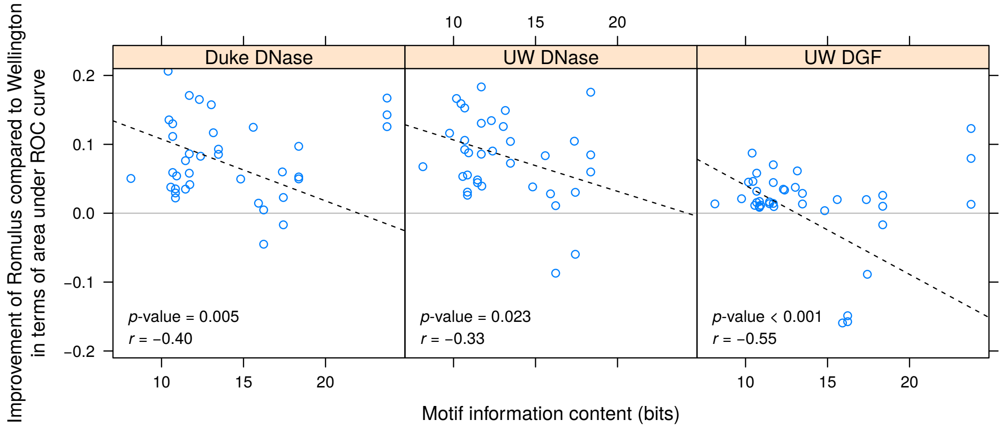

Supplementary Figure 5. Improvement of Romulus compared to Wellington in terms of area under ROC curve significantly correlates with motif information content. All the cell lines (A549, HepG2 and K562) were considered jointly here. Pearson correlation values and p-values were calculated after excluding the outliers with information content above 20 bits.

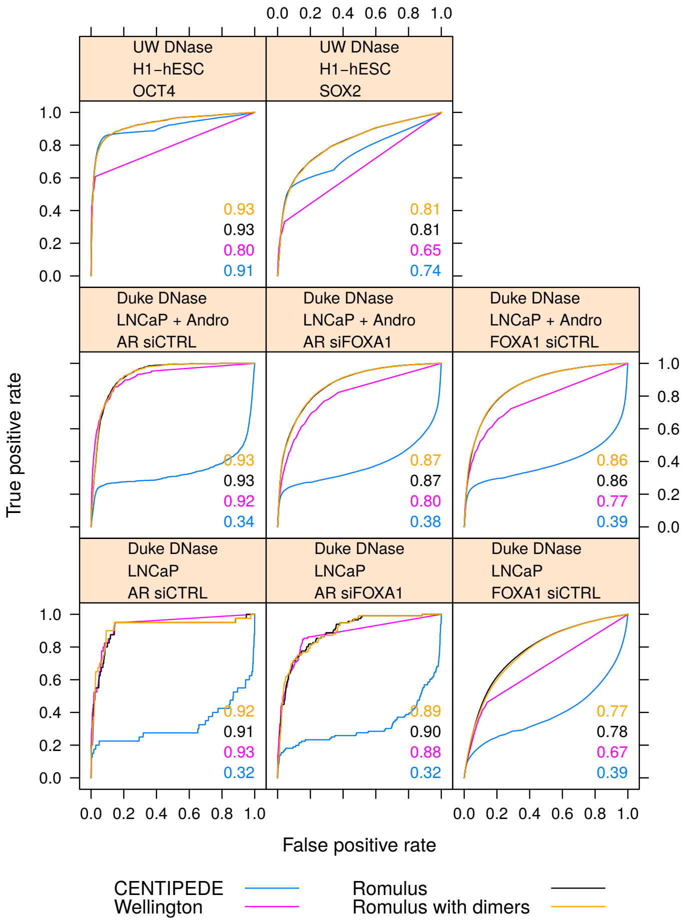

Supplementary Figure 6. Receiver Operating Characteristic curves for the known dimers. The TF in focus (OCT4, SOX2, AR or FOXA1), DNase-seq data source (UW or Duke), and conditions are indicated. +Andro, androgen stimulated cells. siFOXA1, silenced FOXA1. siCTRL, control.

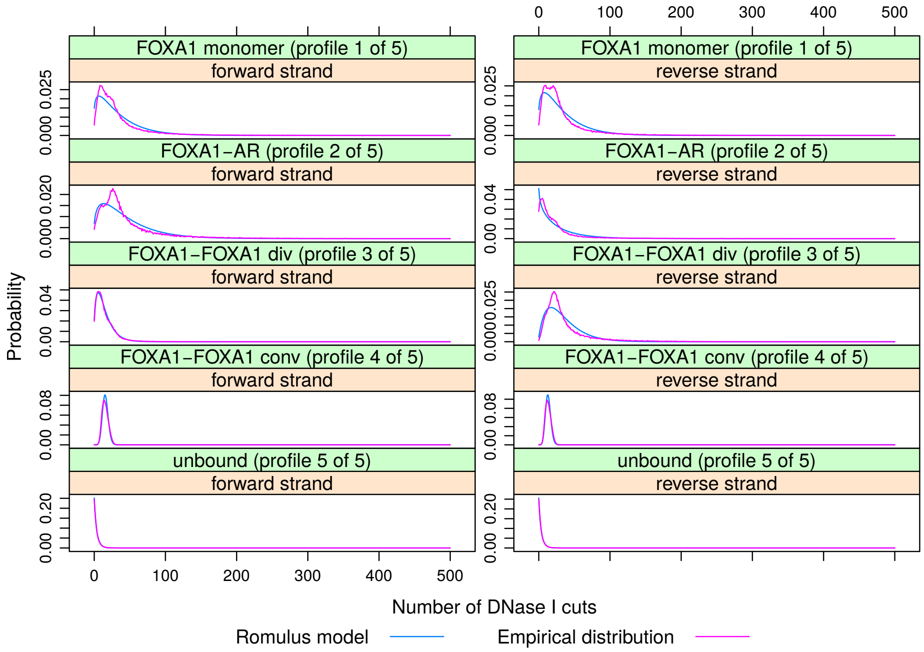

Supplementary Figure 7. Distribution of the number of DNase I cuts learned by Romulus for FOXA1 and its dimers in androgen-stimulated LNCaP cells. The curves show the Romulus negative binomial model and the empirical distribution which is fitted to. *Left:* forward strand cuts, *right:* reverse strand cuts.

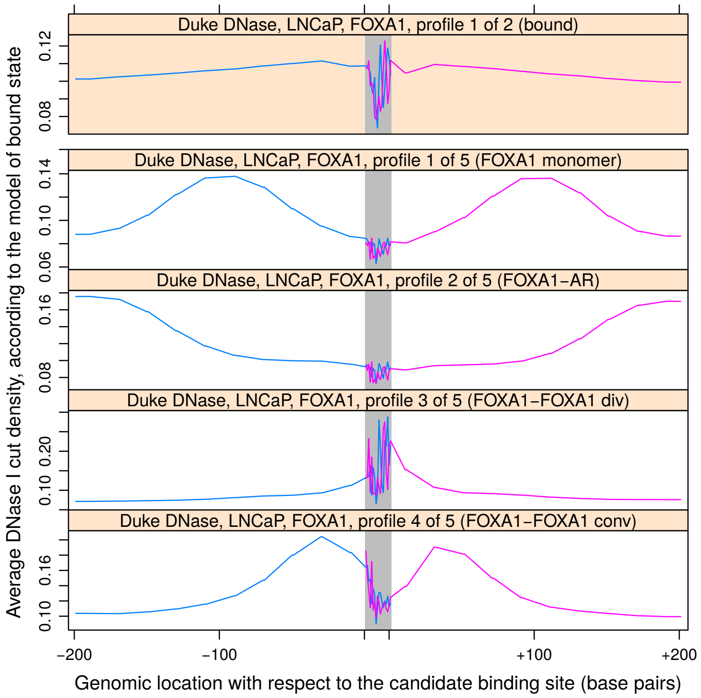

Supplementary Figure 8. Multinomial components of the models learned by Romulus for FOXA1 and its dimers in androgen-stimulated LNCaP cells. The first panel (blue background) corresponds to the model with no dimer binding modes, and is shown here only for comparison. The other panels correspond to all the binding modes in the model allowing for dimerization. The unbound mode always follows the uniform distribution of cuts. The forward strand cuts *(blue line)* are considered only upstream and within the binding site, while the reverse strand cuts *(violet line)* are considered only within the binding site and downstream.

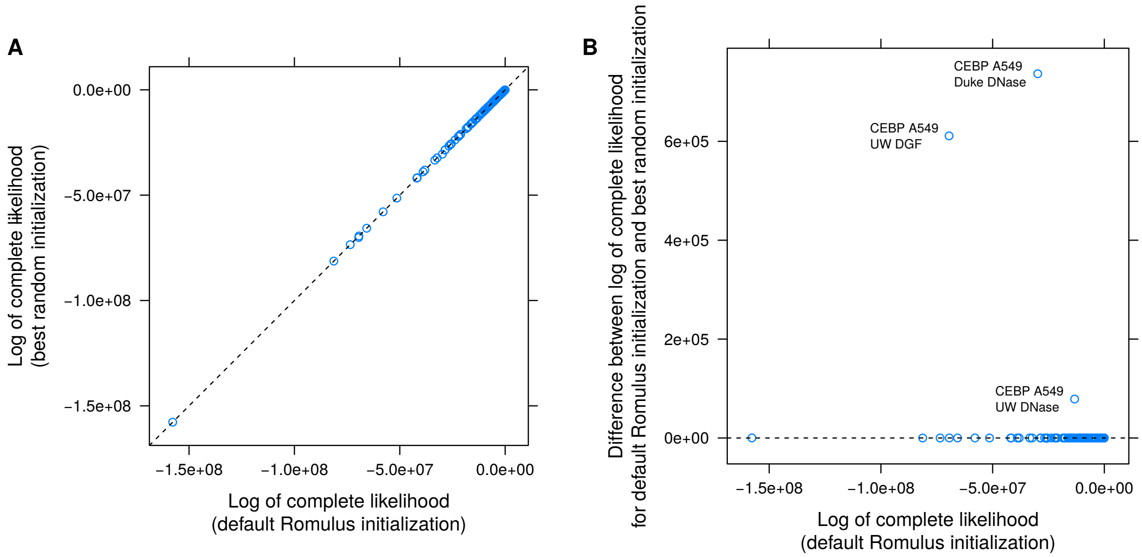

Supplementary Figure 9. (A) Logarithm of complete likelihood of Romulus parameters obtained using the default initialization procedure (x axis) compared to the highest complete likelihood obtained in 10 random initializations (y axis). (B) As above, but compared to the difference in complete likelihood between the default initialization and best random initialization. In total, 117 data points are shown in each panel, each point representing a combination of TF, cell type and DNase I data source.

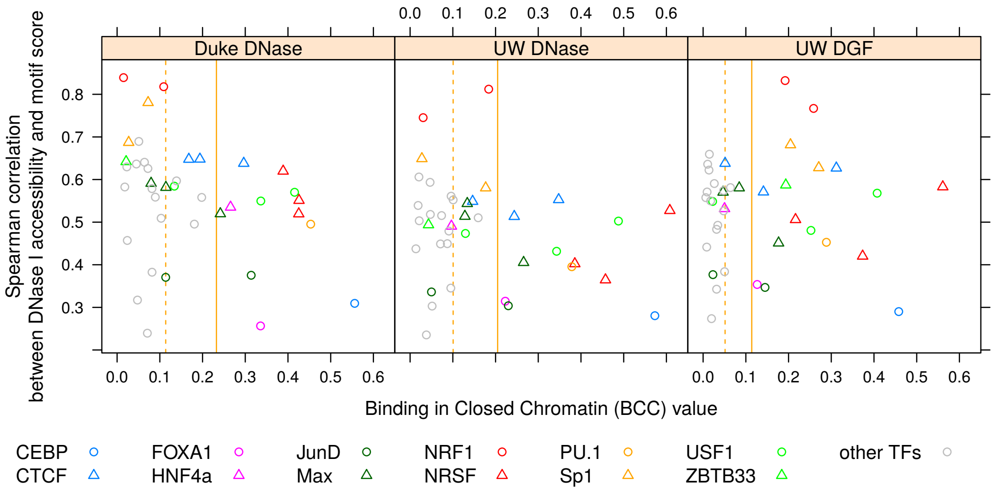

Supplementary Figure 10. Binding in Closed Chromatin (BCC) values (x axis) compared to the Spearman correlation between DNase I accessibility and motif score (y axis). The values are shown for each combination of TF, DNase-seq data source and cell type. For each DNase-seq data source, dashed vertical line indicates the mean, and solid vertical line indicates the threshold of one standard deviation above the mean. In color are shown the TFs having a BCC value, in at least one case, more than one standard deviation above the mean.

Supplementary Tables

Dataset		Number of reads		
Name	Genome Browser track	A549	HepG2	K562
Duke DNase	OpenChromDnase	51.6 M	13.6 M	80.8 M
UW DNase	UwDnase	33.3 M	22.1 M	35.8 M
UW DGF	UwDgf	350.6 M	168.9 M	180.0 M

Supplementary Table 1. Numbers of reads in DNase-seq datasets used. Three ENCODE cell lines were considered: A549, HepG2 and K562. UW, University of Washington; DGF, Digital Genomic Footprinting.

Cell type	Transcription factor	ChIP-seq peaks			Motif instances		
		total	with motif	without motif	overlapping ChIP-seq peaks	outside ChIP-seq peaks	ENCODE narrowPeak filename
K562	ATF3	16 011	2 162	13 849	4 298	160 472	HaibK562Atf3V0416101
K562	c-Myc	5 023	2 098	2 925	4 331	509 454	SydhK562Cmyc
K562	CTCF	56 058	25 788	30 270	26 432	41 170	UtaK562Ctcf
K562	JunD	26 674	2 600	24 074	5 070	112 079	UchicagoK562Ejund
K562	Max	46 171	16 419	29 752	34 226	1 131 646	HaibK562MaxV0416102
K562	NFE2	2 637	1 619	1 018	1 750	50 360	SydhK562Nfe2
K562	NRF1	4 211	2 609	1 602	5 960	20 440	SydhK562Nrf1Iggrab
K562	NRSF	15 849	2 055	13 794	2 112	2 750	HaibK562NrsfV0416102
K562	PU.1	28 677	18 514	10 163	20 262	549 324	HaibK562Pu1Pcr1x
K562	Sp1	7 206	2 830	4 376	4 861	137 043	HaibK562Sp1Pcr1x
K562	USF1	18 521	12 431	6 090	23 808	524 887	HaibK562Usf1V0416101
A549	ATF3	6 580	308	6 272	636	164 134	HaibA549Atf3V0422111Etoh02
A549	bHLHE40	3 123	1 225	1 898	2 667	254 098	SydhA549Bhlhe40Iggrab
A549	CEBP	38 845	25 305	13 540	46 517	1 722 846	SydhA549CebpbIggrab
A549	CTCF	45 732	23 536	22 196	24 289	43 313	UwA549Ctcf
A549	ELF1	8 611	5 075	3 536	6 937	348 641	HaibA549Elf1V0422111Etoh02
A549	ETS1	5 525	2 564	2 961	3 466	1 145 420	HaibA549Ets1V0422111Etoh02
A549	GABP	12 348	7 196	5 152	9 396	871 718	HaibA549GabpV0422111Etoh02
A549	Max	9 881	3 982	5 899	8 965	1 156 907	SydhA549MaxIggrab
A549	NRSF	11 970	1 938	10 032	1 861	3 001	HaibA549NrsfV0422111Etoh02
A549	USF1	8 004	4 710	3 294	9 452	539 243	HaibA549Usf1V0422111Etoh02
A549	YY1	10 259	2 148	8 111	2 079	52 873	HaibA549Yy1cV0422111Etoh02
A549	ZBTB33	7 152	626	6 526	1 052	14 443	HaibA549Zbtb33V0422111Etoh02
HepG2	ATF3	3 291	1 132	2 159	2 392	162 378	HaibHepg2Atf3V0416101
HepG2	c-Myc	4 413	1 762	2 651	3 558	510 227	UtaHepg2Cmyc
HepG2	CTCF	55 778	26 856	28 922	27 655	39 947	HaibHepg2Ctcfsc5916V0416101
HepG2	FOXA1	40 989	29 356	11 633	76 105	6 363 288	HaibHepg2Foxa2sc6554V0416101
HepG2	HNF4a	20 805	10 913	9 892	12 889	519 223	HaibHepg2Hnf4asc8987V0416101
HepG2	JunD	21 614	866	20 748	1 632	115 517	HaibHepg2JundPcr1x
HepG2	Max	11 854	4 707	7 147	10 726	1 155 146	SydhHepg2MaxIggrab
HepG2	MYB	17 898	8 016	9 882	10 306	2 389 507	HaibHepg2Mybl2sc81192V0422111
HepG2	NRF1	1 902	1 635	267	4 132	22 268	SydhHepg2Nrf1Iggrab
HepG2	NRSF	12 828	1 686	11 142	1 743	3 119	HaibHepg2NrsfV0416101
HepG2	RXR	17 063	6 976	10 087	9 044	1 265 842	HaibHepg2RxraPcr1x
HepG2	Sp1	25 477	3 599	21 878	6 087	135 817	HaibHepg2Sp1Pcr1x
HepG2	Srebp1a	2 585	293	2 292	307	327 401	SydhHepg2Srebp1Insln
HepG2	TBP	13 806	2 490	11 316	3 798	3 136 778	SydhHepg2TbpIggrab
HepG2	TR4	2 953	660	2 293	836	88 251	SydhHepg2Tr4Ucd
HepG2	USF1	21 890	14 809	7 081	27 503	521 192	HaibHepg2Usf1Pcr1x
Total		670 214	283 494	386 720	449 140	26 312 163	
Percentage			42.3%	57.7%	1.7%	98.3%	

Supplementary Table 2. ChIP-seq datasets used as the reference classification of the candidate binding sites. These datasets were generated by the ENCODE Analysis Working Group (AWG) using a uniform processing pipeline. The narrowPeak filenames follow the pattern "wgEncodeAwgTfbs...UniPk.narrowPeak.gz", where only the changing "..." part is given above.

Transcription factor	Motif identifier(s) in HOMER
ATF3	ATF3(bZIP)/K562-ATF3
bHLHE40	bHLHE40(HLH)/HepG2-BHLHE40
CEBP	CEBP(bZIP)/CEBPb
c-Myc	c-Myc(HLH)/LNCAP-cMyc
CTCF	CTCF(Zf)/CD4+-CTCF
ELF1	ELF1(ETS)/Jurkat-ELF1
ETS1	ETS1(ETS)/Jurkat-ETS1
FOXA1	FOXA1(Forkhead)/LNCAP-FOXA1
	FOXA1(Forkhead)/MCF7-FOXA1
GABP	GABPA(ETS)/Jurkat-GABPa
HNF4a	HNF4a(NR/DR1)/HepG2-HNF4a
JunD	JunD(bZIP)/K562-JunD
Max	Max(HLH)/K562-Max
MYB	MYB(HTH)/ERMYB-Myb-ChIPSeq(GSE22095)
NFE2	NF-E2(bZIP)/K562-NFE2
NRF1	NRF1(NRF)/MCF7-NRF1
NRSF	REST-NRSF(Zf)/Jurkat-NRSF
PU.1	PU.1(ETS)/ThioMac-PU.1
RXR	RXR(NR/DR1)/3T3L1-RXR
Sp1	Sp1(Zf)/Promoter
Srebp1a	Srebp1a(HLH)/HepG2-Srebp1a
TBP	TATA-Box(TBP)/Promoter
TR4	TR4(NR/DR1)/Hela-TR4
USF1	USF1(HLH)/GM12878-Usf1
YY1	YY1(Zf)/Promoter
ZBTB33	ZBTB33/GM12878-ZBTB33

Supplementary Table 3. HOMER motif identifiers used for each TF. The corresponding sets of significant motif instances were downloaded from HOMER. For FOXA1, two motifs were used, and the union of corresponding two sets of motif instances was taken. For all the other TFs, one motif was used.

			ChIP-seq peaks			Motif instances		
Cell type	Transcription factor	Treatment	total	with motif	without motif	overlapping ChIP-seq peaks	outside ChIP-seq peaks	Source
H1-hESC	OCT4 (POU5F1)		6 289	3 018	3 271	6 913	1 347 572	GSM447582
H1-hESC	SOX2		20 035	16 645	3 390	78 824	1 282 668	GSM456570
LNCaP + Andro	AR	siCTRL	3 743	499	3 244	1 031	268 898	GSM686917
LNCaP + Andro	AR	siFOXA1	14 400	4 885	9 515	24 097	245 832	GSM686920
LNCaP + Andro	FOXA1	siCTRL	36 344	14 656	21 688	99 797	1 856 212	GSM686926
LNCaP	AR	siCTRL	6 028	31	5 997	40	269 889	GSM686914
LNCaP	AR	siFOXA1	4 410	66	4 344	116	269 813	GSM686919
LNCaP	FOXA1	siCTRL	46 299	21 418	24 881	158 319	1 797 690	GSM686925

Supplementary Table 4. ChIP-seq datasets used as the reference classification of the candidate binding sites for dimerizing transcription factors. Motif instances were identified using TRANSFAC motifs M00795 (OCT4), M01247 (SOX2), M00960 (AR) and M01012 (FOXA1), using motif score threshold that provides 80% sensitivity. Last column indicates Gene Expression Omnibus identifier.

Supplementary Methods

1 Prior probabilities of TF binding

To model the prior probabilities, we apply a logistic model against the unbound "pivot" case of $Z_i = 0$:

$$\frac{P(Z_i = k)}{P(Z_i = 0)} = \exp\left(\beta_0^{(k)} + \sum_j \beta_j^{(k)} \gamma_j^{(k)} x_i^{(j)}\right), \tag{1}$$

where $k = 0$ indicates no binding, $k = 1$ refers to binding as monomer, and $k = 2, \ldots, K+1$ refer to the respective cooperative binding modes. This way, we have $K + 1$ outcomes separately regressed against the pivot outcome $Z_i = 0$.

For clarity of presentation, we impose an additional constraint such that $\gamma_j^{(k)} = 0$ implies $\beta_j^{(k)} = 0$. In other words, $\beta_j^{(k)} = 0$ for the partner motifs not involved in k-th binding mode. We can now explicitly formulate $P(Z_i = 0)$ by summing up Equation 1 for $k = 1, \ldots, K + 1$:

$$\frac{\sum_{k=1}^{K+1} P(Z_i = k)}{P(Z_i = 0)} = \sum_{k=1}^{K+1} \exp\left(\beta_0^{(k)} + \sum_j \beta_j^{(k)} \gamma_j^{(k)} x_i^{(j)}\right) \tag{2}$$

$$\frac{1 - P(Z_i = 0)}{P(Z_i = 0)} = \sum_{k=1}^{K+1} \exp\left(\beta_0^{(k)} + \sum_j \beta_j^{(k)} \gamma_j^{(k)} x_i^{(j)}\right) \tag{3}$$

$$P(Z_i = 0) = \frac{1}{1 + \sum_{k=1}^{K+1} \exp\left(\beta_0^{(k)} + \sum_j \beta_j^{(k)} \gamma_j^{(k)} x_i^{(j)}\right)}. \tag{4}$$

Applying the above to Equation 1, we obtain an explicit formulation for all the probabilities $P(Z_i = k)$ where $k > 0$:

$$P(Z_i = k) = \frac{\exp\left(\beta_0^{(k)} + \sum_j \beta_j^{(k)} \gamma_j^{(k)} x_i^{(j)}\right)}{1 + \sum_{l=1}^{K+1} \exp\left(\beta_0^{(l)} + \sum_j \beta_j^{(l)} \gamma_j^{(l)} x_i^{(j)}\right)}. \tag{5}$$

2 Chromatin state component

Our primary interest is

$$p_i = \sum_{k=1}^{K+1} P(Z_i = k \mid X_i) = 1 - P(Z_i = 0 \mid X_i), \tag{6}$$

i.e. the probability of the motif instance i to be bound in any binding mode.

Taking the complement and following the Bayes' theorem, we get

$$1 - p_i = P(Z_i = 0 \mid X_i) = \frac{P(X_i \mid Z_i = 0)P(Z_i = 0)}{\sum_{k=0}^{K+1} P(X_i \mid Z_i = k)P(Z_i = k)} \tag{7}$$

$$\frac{1}{1 - p_i} = \sum_{k=0}^{K+1} \frac{P(X_i \mid Z_i = k)P(Z_i = k)}{P(X_i \mid Z_i = 0)P(Z_i = 0)} = 1 + \sum_{k=1}^{K+1} \frac{P(X_i \mid Z_i = k)P(Z_i = k)}{P(X_i \mid Z_i = 0)P(Z_i = 0)} \tag{8}$$

$$\frac{p_i}{1 - p_i} = \sum_{k=1}^{K+1} \frac{P(X_i \mid Z_i = k)P(Z_i = k)}{P(X_i \mid Z_i = 0)P(Z_i = 0)}. \tag{9}$$

We make a simplifying assumption that all the chromatin state data included in the model are independent, given its binding state Z_i. Hence, the conditional probability $P(X_i \mid Z_i = k)$ is a product of the corresponding conditional probabilities:

$$P(X_i \mid Z_i = k) = P((\text{DNase}^+_{i,j})_j \mid Z_i = k) \cdot P((\text{DNase}^-_{i,j})_j \mid Z_i = k) \cdot \ldots . \tag{10}$$

For brevity, we discuss the formulas for the forward strand DNase I component only; they are analogous for the reverse strand and for other types of data. The negative binomial component in binding mode k quantifies the total number of DNase I cuts on the forward strand

$$\text{DNaseSum}^+_i = \sum_j \text{DNase}^+_{i,j} \tag{11}$$

and is naturally parametrized by the success probability $p^{+(k)} \in (0, 1)$ and the real-valued number of failures $r^{+(k)} > 0$.

The multinomial component quantifies the probability of a particular spatial distribution of the total number of DNase I cuts on a given strand. For each binding mode k and positional data type (e.g. DNase I cuts on forward strand), we divide the positions j into one or more bins. Let us denote by $\text{DNaseBin}^{+(k)}_j$ the bin number for position j in binding mode k. For clarity, let us assume that the bins are numbered by positive integers. In this study, we take 20 bp long bins outside the motif site, and single-basepair bins within the motif site. Moreover, for the unbound mode ($k = 0$) we put all the positions in a single bin:

$$\text{DNaseBin}^{+(k)}_j = \begin{cases} 1 & \text{for } k = 0 \text{ and any } j \\ \lceil j/20 \rceil & \text{for } k > 0 \text{ and } j = 1, \ldots, 200 \\ 190 - j & \text{for } k > 0 \text{ and } j = 201, \ldots, 200 + L. \end{cases} \tag{12}$$

Note that binding modes may differ in the way the positions are split into bins.

For a given binding mode k, we associate a free parameter $\lambda^{+(k)}_b$ with each bin $b = 1, \ldots, B^{+(k)}$. However, for the multinomial distribution we must provide a vector of probabilities covering every single position in the vicinity of the motif instance. Hence, we calculate the actual multinomial coefficients $\tilde{\lambda}^{+(k)}_j$ by taking $\lambda^{+(k)}_b$ for $b = \text{DNaseBin}^{+(k)}_j$ and normalizing $\lambda^{+(k)}_b$ so that $\sum_j \tilde{\lambda}^{+(k)}_j = 1$. By definition, the multinomial coefficients $\tilde{\lambda}^{+(0)}_j$ for the unbound state are equal, i.e. there is no positional preference for DNase I cuts in the null model.

The joint probability of the DNase I positional data is obtained by the superposition of the negative binomial and multinomial components:

$$
\begin{aligned}
&P((\mathrm{DNase}^{+}_{i,j})_j \mid Z_i = k) \\
&\qquad = \mathrm{NegativeBinomial}\left(\mathrm{DNaseSum}^{+}_{i} \mid p^{+(k)}, r^{+(k)}\right) \\
&\qquad\qquad \cdot \mathrm{Multinomial}\left((\mathrm{DNase}^{+}_{i,j})_j \mid \mathrm{DNaseSum}^{+}_{i}, (\lambda^{+(k)}_{b})_b\right). \qquad (13)
\end{aligned}
$$

Now we can explicitly formulate the probabilities:

$$
\begin{aligned}
&\mathrm{NegativeBinomial}\left(\mathrm{DNaseSum}^{+}_{i} \mid p^{+(k)}, r^{+(k)}\right) \\
&\qquad = \frac{\Gamma\left(r^{+(k)} + \mathrm{DNaseSum}^{+}_{i}\right)}{\Gamma\left(\mathrm{DNaseSum}^{+}_{i} + 1\right)\,\Gamma(r^{+(k)})} (p^{+(k)})^{r^{+(k)}} (1 - p^{+(k)})^{\mathrm{DNaseSum}^{+}_{i}} \qquad (14)
\end{aligned}
$$

$$
\begin{aligned}
&\mathrm{Multinomial}\left((\mathrm{DNase}^{+}_{i,j})_j \mid \mathrm{DNaseSum}^{+}_{i}, (\lambda^{+(k)}_{b})_b\right) \\
&\qquad = \mathrm{DNaseSum}^{+}_{i}! \prod_j \frac{\left(\tilde{\lambda}^{+(k)}_{j}\right)^{\mathrm{DNase}^{+}_{i,j}}}{\mathrm{DNase}^{+}_{i,j}!} \\
&\qquad = \Gamma\left(\mathrm{DNaseSum}^{+}_{i} + 1\right) \prod_j \frac{\left(\tilde{\lambda}^{+(k)}_{j}\right)^{\mathrm{DNase}^{+}_{i,j}}}{\Gamma\left(\mathrm{DNase}^{+}_{i,j} + 1\right)}, \qquad (15)
\end{aligned}
$$

where Γ is the standard gamma function, i.e. a continuous extension of the factorial function.

3 Expectation-Maximization approach

To estimate the model parameters

$$
\Theta = \left((\beta^{(k)}_{j})_{j,k}, (p^{+(k)})_k, (p^{-(k)})_k, (r^{+(k)})_k, (r^{-(k)})_k, (\lambda^{+(k)}_{b})_{b,k}, (\lambda^{-(k)}_{b})_{b,k}\right), \qquad (16)
$$

we apply the Expectation-Maximization approach. We use a common technique: instead of maximizing the likelihood function

$$
L(\Theta) = \prod_i P(X_i \mid \Theta) \qquad (17)
$$

with unknown latent state, we maximize the complete likelihood function

$$
L_C(\Theta) = \prod_i P(X_i, Z_i \mid \Theta) = \prod_i P(X_i \mid Z_i, \Theta) P(Z_i \mid \Theta), \qquad (18)
$$

which is more tractable.

The complete likelihood function, as stated above, is defined only for $Z_i = 0, \ldots, K+1$. However, we may rewrite it using indicator functions $Z^{(k)}_{i}$ such that $Z^{(k)}_{i} = 1$ if $Z_i = k$ and

$Z_i^{(k)} = 0$ otherwise:

$$L_C(\Theta) = \prod_i \prod_{k=0}^{K+1} P(X_i \mid Z_i = k, \Theta)^{Z_i^{(k)}} P(Z_i = k \mid \Theta)^{Z_i^{(k)}}. \tag{19}$$

Let us denote by $\langle Z_i^{(k)} \rangle$ the expected value of $Z_i^{(k)}$. It holds that $\langle Z_i^{(k)} \rangle = P(Z_i = k)$. Taking the expected value of $L_C(\Theta)$ with respect to all $Z_i^{(k)}$, we obtain a real-domain function of Θ:

$$\langle L_C(\Theta) \rangle = \prod_i \prod_{k=0}^{K+1} P(X_i \mid Z_i = k, \Theta)^{\langle Z_i^{(k)} \rangle} P(Z_i = k \mid \Theta)^{\langle Z_i^{(k)} \rangle}. \tag{20}$$

The formulas will easier to manipulate after taking the logarithm:

$$\log\langle L_C(\Theta) \rangle = \overbrace{\sum_i \sum_{k=0}^{K+1} \langle Z_i^{(k)} \rangle \log P(X_i \mid Z_i = k, \Theta)}^{L_A(\Theta)} + \underbrace{\sum_i \sum_{k=0}^{K+1} \langle Z_i^{(k)} \rangle \log P(Z_i = k \mid \Theta)}_{L_B(\Theta)}. \tag{21}$$

Our goal is to maximize the (log-transformed) expected value of the complete likelihood function L_C. Note that the value of the first component, L_A, depends on $(p^{+(k)})_k$, $(p^{-(k)})_k$, $(r^{+(k)})_k$, $(r^{-(k)})_k$, $(\lambda_b^{+(k)})_{b,k}$ and $(\lambda_b^{-(k)})_{b,k}$, while the value of the second component, L_B, depends only on the parameters in Θ not listed previously, namely on $(\beta_j^{(k)})_{j,k}$. Therefore, we can maximize L_A and L_B separately.

We found no closed-form solution for $\beta_j^{(k)}$ that maximizes $L_B(\Theta)$, hence we apply the Broyden-Fletcher-Goldfarb-Shanno (BFGS) numerical optimization procedure here. This method uses the function values and gradients to build up a representation of the surface to be maximized. Substituting Equation 5 to the definition of L_B, we get:

$$L_B(\Theta) = \sum_i \sum_{k=0}^{K+1} \langle Z_i^{(k)} \rangle \left(\beta_0^{(k)} + \sum_j \beta_j^{(k)} \gamma_j^{(k)} x_i^{(j)} \right) - \sum_i \log\left(1 + \sum_{l=1}^{K+1} \exp\left(\beta_0^{(l)} + \sum_j \beta_j^{(l)} \gamma_j^{(l)} x_i^{(j)} \right)\right) \sum_{k=0}^{K+1} \langle Z_i^{(k)} \rangle. \tag{22}$$

Note that the last factor, $\sum_{k=0}^{K+1} \langle Z_i^{(k)} \rangle$, is equal to 1 and may thus be omitted. Differentiating L_B with respect to $\beta_j^{(k)}$, we get:

$$\frac{\partial L_B}{\partial \beta_j^{(k)}} = \sum_i \langle Z_i^{(k)} \rangle \gamma_j^{(k)} x_i^{(j)} - \sum_i \frac{\exp\left(\beta_0^{(k)} + \sum_j \beta_j^{(k)} \gamma_j^{(k)} x_i^{(j)}\right) \gamma_j^{(k)} x_i^{(j)}}{1 + \sum_{l=1}^{K+1} \exp\left(\beta_0^{(l)} + \sum_j \beta_j^{(l)} \gamma_j^{(l)} x_i^{(j)}\right)}. \tag{23}$$

Now let us focus on the other component of $\log\langle L_C(\Theta)\rangle$, i.e. L_A. For clarity, let us assume that DNase-seq data is the only kind of positional data provided. The derivations follow analogously for any other independent positional datasets. Substituting Equations 10 to the definition of L_B in Equation 21, we get:

$$L_A(\Theta) = \overbrace{\sum_i \sum_{k=0}^{K+1} \langle Z_i^{(k)} \rangle \log P((\mathrm{DNase}_{i,j}^{+})_j \mid Z_i = k)}^{L_A^{+}(\Theta)} \\ + \underbrace{\sum_i \sum_{k=0}^{K+1} \langle Z_i^{(k)} \rangle \log P((\mathrm{DNase}_{i,j}^{-})_j \mid Z_i = k)}_{L_A^{-}(\Theta)} . \quad (24)$$

The two components, L_A^+ and L_A^-, depend on distinct sets of parameters in the same manner. Hence, we can maximize them separately. Without loss of generality, we will discuss the optimization procedure for L_A^+. From Equations 13 to 15, we have:

$$\begin{aligned} L_A^+(\Theta) = \sum_i \sum_{k=0}^{K+1} \langle Z_i^{(k)} \rangle \log \Bigg(& \frac{\Gamma\big(r^{+(k)} + \mathrm{DNaseSum}_i^+\big)}{\Gamma\big(\mathrm{DNaseSum}_i^+ + 1\big)\ \Gamma(r^{+(k)})} \\ & \cdot (p^{+(k)})^{r^{+(k)}} (1 - p^{+(k)})^{\mathrm{DNaseSum}_i^+} \Bigg) \\ + \sum_i \sum_{k=0}^{K+1} \langle Z_i^{(k)} \rangle \log & \left(\Gamma\big(\mathrm{DNaseSum}_i^+ + 1\big) \prod_j \frac{\left(\tilde{\lambda}_j^{+(k)}\right)^{\mathrm{DNase}_{i,j}^+}}{\Gamma\big(\mathrm{DNase}_{i,j}^+ + 1\big)} \right) \\ = \sum_i \sum_{k=0}^{K+1} & \langle Z_i^{(k)} \rangle \log\Gamma\big(r^{+(k)} + \mathrm{DNaseSum}_i^+\big) \\ - \sum_i \sum_{k=0}^{K+1} \langle Z_i^{(k)} \rangle \log\Gamma\big(\mathrm{DNaseSum}_i^+ + 1\big) & - \sum_i \sum_{k=0}^{K+1} \langle Z_i^{(k)} \rangle \log\Gamma(r^{+(k)}) \\ + \sum_i \sum_{k=0}^{K+1} \langle Z_i^{(k)} \rangle r^{+(k)} \log(p^{+(k)}) & + \sum_i \sum_{k=0}^{K+1} \langle Z_i^{(k)} \rangle \mathrm{DNaseSum}_i^+ \log(1 - p^{+(k)}) \\ + \sum_i \sum_{k=0}^{K+1} & \langle Z_i^{(k)} \rangle \log\Gamma\big(\mathrm{DNaseSum}_i^+ + 1\big) \\ + \sum_i \sum_{k=0}^{K+1} \langle Z_i^{(k)} \rangle \sum_j \mathrm{DNase}_{i,j}^+ \log\left(\tilde{\lambda}_j^{+(k)}\right) & - \sum_i \sum_{k=0}^{K+1} \langle Z_i^{(k)} \rangle \sum_j \log\Gamma\big(\mathrm{DNase}_{i,j}^+ + 1\big). \end{aligned} \quad (25)$$

Eliminating the additive inverse terms and noting that $\sum_{k=0}^{K+1}\langle Z_i^{(k)}\rangle = 1$, we get:

$$L_A^+(\Theta) = \sum_i \sum_{k=0}^{K+1} \langle Z_i^{(k)}\rangle \log\Gamma\big(r^{+(k)} + \mathrm{DNaseSum}_i^+\big) - \sum_i \sum_{k=0}^{K+1} \langle Z_i^{(k)}\rangle \log\Gamma(r^{+(k)})$$
$$+ \sum_i \sum_{k=0}^{K+1} \langle Z_i^{(k)}\rangle r^{+(k)} \log(p^{+(k)}) + \sum_i \sum_{k=0}^{K+1} \langle Z_i^{(k)}\rangle \mathrm{DNaseSum}_i^+ \log(1-p^{+(k)})$$
$$+ \sum_i \sum_{k=0}^{K+1} \langle Z_i^{(k)}\rangle \sum_j \mathrm{DNase}_{i,j}^+ \log\left(\tilde{\lambda}_j^{+(k)}\right) - \sum_i \sum_j \log\Gamma\big(\mathrm{DNase}_{i,j}^+ + 1\big). \quad (26)$$

Note that only the first three summands depend on $(r^{+(k)})_k$, only the third and fourth depends on $(p^{+(k)})_k$, and only the fifth depends on the parameters $(\lambda_b^{+(k)})_{b,k}$, which give rise to $\left(\tilde{\lambda}_j^{+(k)}\right)_{j,k}$. Hence, we may find the values of $(\lambda_b^{+(k)})_{b,k}$ that maximize L_A independently of the other parameters. We need to maximize

$$\sum_{k=0}^{K+1} \sum_j \log\left(\tilde{\lambda}_j^{+(k)}\right) \sum_i \langle Z_i^{(k)}\rangle \mathrm{DNase}_{i,j}^+ \quad (27)$$

subject to the constraint $\sum_j \tilde{\lambda}_j^{+(k)} = 1$ for each k. Since k-th element in the sum above depends only on $\left(\tilde{\lambda}_j^{+(k)}\right)_j$ and consequently only on $(\lambda_j^{+(k)})_j$, we can maximize each element of the sum independently. We use a common technique, and for a given k maximize the expression

$$\sum_j \log\left(\tilde{\lambda}_j^{+(k)}\right) \sum_i \langle Z_i^{(k)}\rangle \mathrm{DNase}_{i,j}^+ + L \cdot \Big(1 - \sum_j \tilde{\lambda}_j^{+(k)}\Big), \quad (28)$$

where L is called the Lagrange multiplier.

Let us recall that the multinomial coefficients $\tilde{\lambda}_j^{+(k)}$ are equal to the corresponding parameters $\lambda_b^{+(k)}$ such that $b = \mathrm{DNaseBin}_j^{+(k)}$. Now let us fix the bin b and define the set J_b grouping all the positions j falling within bin b:

$$J_b = \left\{ j \colon \mathrm{DNaseBin}_j^{+(k)} = b \right\}. \quad (29)$$

Differentiating Formula 28 with respect to $\lambda_b^{+(k)}$ and setting the derivative equal to zero, we get:

$$0 = \sum_{j \in J_b} \frac{1}{\lambda_b^{+(k)}} \sum_i \langle Z_i^{(k)}\rangle \mathrm{DNase}_{i,j}^+ - L \cdot |J_b|. \quad (30)$$

Note that the above is a decreasing function of $\lambda_b^{+(k)}$, hence we capture a local maximum here. Hence,

$$L \cdot |J_b| \lambda_b^{+(k)} = \sum_{j \in J_b} \sum_i \langle Z_i^{(k)}\rangle \mathrm{DNase}_{i,j}^+ \quad (31)$$

and summing this equation over all $b = 1, \ldots, B^{+(k)}$, we get

$$L \cdot \sum_{b=1}^{B^{+(k)}} |J_b| \lambda_b^{+(k)} = \sum_{b=1}^{B^{+(k)}} \sum_{j \in J_b} \sum_i \langle Z_i^{(k)}\rangle \mathrm{DNase}_{i,j}^+. \quad (32)$$

We should now note that

$$1 = \sum_j \tilde{\lambda}_j^{+(k)} = \sum_{b=1}^{B^{+(k)}} \sum_{j \in J_b} \tilde{\lambda}_j^{+(k)} = \sum_{b=1}^{B^{+(k)}} |J_b| \lambda_b^{+(k)}. \tag{33}$$

Now Equation 32 becomes

$$L = \sum_{b=1}^{B^{+(k)}} \sum_{j \in J_b} \sum_i \langle Z_i^{(k)} \rangle \mathrm{DNase}_{i,j}^{+}, \tag{34}$$

and substituting the above into Equation 31, we obtain the desired solution:

$$\lambda_b^{+(k)} = \frac{\sum_{j \in J_b} \sum_i \langle Z_i^{(k)} \rangle \mathrm{DNase}_{i,j}^{+}}{|J_b| \sum_{c=1}^{B^{+(k)}} \sum_{j \in J_c} \sum_i \langle Z_i^{(k)} \rangle \mathrm{DNase}_{i,j}^{+}}. \tag{35}$$

To increase the robustness of the model, we employ a shrinkage estimator of the parameters $(\lambda_b^{+(k)})_{b,k}$. For each b and k, we take the regularized estimator

$$\delta \lambda_b^{+(k)} + (1-\delta) \frac{|J_b|}{\sum_b |J_b|}, \tag{36}$$

where the mixing parameter δ is by default equal to 0.5.

Now we will find the values of $(p^{+(k)})_k$ that maximize L_A independently of the other parameters. Differentiating Equation 26 with respect to $p^{+(k)}$ and setting the derivative equal to zero, we obtain the closed form for $p^{+(k)}$:

$$0 = \frac{\partial L_A^+}{\partial p^{+(k)}} = \sum_i \langle Z_i^{(k)} \rangle r^{+(k)} \frac{1}{p^{+(k)}} - \sum_i \langle Z_i^{(k)} \rangle \mathrm{DNaseSum}_i^{+} \frac{1}{1 - p^{+(k)}} \tag{37}$$

$$p^{+(k)} \sum_i \langle Z_i^{(k)} \rangle \mathrm{DNaseSum}_i^{+} = (1 - p^{+(k)}) \sum_i \langle Z_i^{(k)} \rangle r^{+(k)} \tag{38}$$

$$p^{+(k)} = \frac{\sum_i \langle Z_i^{(k)} \rangle r^{+(k)}}{\sum_i \langle Z_i^{(k)} \rangle \mathrm{DNaseSum}_i^{+} + \sum_i \langle Z_i^{(k)} \rangle r^{+(k)}}. \tag{39}$$

Again, the above is a decreasing function of $p^{+(k)}$, indicating a local maximum here.

It remains to establish the values of $(r^{+(k)})_k$ that maximize L_A. Let us recall that only the first four summands in Equation 26 depend on $(r^{+(k)})_k$ or $(p^{+(k)})_k$. We start with substituting

Equation 39 into these four summands:

$$
\begin{aligned}
L'^{+}_{A}(\Theta) = &\sum_i \sum_{k=0}^{K+1} \langle Z_i^{(k)} \rangle \log \Gamma\big(r^{+(k)} + \text{DNaseSum}_i^{+}\big) \\
&- \sum_i \sum_{k=0}^{K+1} \langle Z_i^{(k)} \rangle \log \Gamma(r^{+(k)}) + \sum_i \sum_{k=0}^{K+1} \langle Z_i^{(k)} \rangle r^{+(k)} \log \left(\sum_l \langle Z_l^{(k)} \rangle r^{+(k)} \right) \\
&- \sum_i \sum_{k=0}^{K+1} \langle Z_i^{(k)} \rangle r^{+(k)} \log \left(\sum_l \langle Z_l^{(k)} \rangle \text{DNaseSum}_l^{+} + \sum_l \langle Z_l^{(k)} \rangle r^{+(k)} \right) \\
&+ \sum_i \sum_{k=0}^{K+1} \langle Z_i^{(k)} \rangle \text{DNaseSum}_i^{+} \log \left(\sum_l \langle Z_l^{(k)} \rangle \text{DNaseSum}_l^{+} \right) \\
&- \sum_i \sum_{k=0}^{K+1} \langle Z_i^{(k)} \rangle \text{DNaseSum}_i^{+} \log \left(\sum_l \langle Z_l^{(k)} \rangle \text{DNaseSum}_l^{+} + \sum_l \langle Z_l^{(k)} \rangle r^{+(k)} \right). \qquad (40)
\end{aligned}
$$

Unfortunately, there seems to be no closed-form solution for $r^{+(k)}$ that maximizes $L'^{+}_{A}(\Theta)$. Here we again apply the BFGS numerical optimization. For brevity, let us introduce the digamma function, $\psi(x) = \frac{\Gamma'(x)}{\Gamma(x)}$. Differentiating L^{+}_{A} with respect to $r^{+(k)}$, we get:

$$
\begin{aligned}
\frac{\partial L^{+}_{A}}{\partial r^{+(k)}} = \frac{\partial L'^{+}_{A}}{\partial r^{+(k)}} = &\sum_i \langle Z_i^{(k)} \rangle \psi\big(r^{+(k)} + \text{DNaseSum}_i^{+}\big) - \sum_i \langle Z_i^{(k)} \rangle \psi(r^{+(k)}) \\
&+ \log \left(\sum_i \langle Z_i^{(k)} \rangle r^{+(k)} \right) \sum_i \langle Z_i^{(k)} \rangle + \sum_i \langle Z_i^{(k)} \rangle \\
&- \log \left(\sum_i \langle Z_i^{(k)} \rangle \text{DNaseSum}_i^{+} + \sum_i \langle Z_i^{(k)} \rangle r^{+(k)} \right) \sum_i \langle Z_i^{(k)} \rangle \\
&- \frac{\sum_i \langle Z_i^{(k)} \rangle}{\sum_i \langle Z_i^{(k)} \rangle \text{DNaseSum}_i^{+} + \sum_i \langle Z_i^{(k)} \rangle r^{+(k)}} \sum_i \langle Z_i^{(k)} \rangle r^{+(k)} \\
&- \frac{\sum_i \langle Z_i^{(k)} \rangle}{\sum_i \langle Z_i^{(k)} \rangle \text{DNaseSum}_i^{+} + \sum_i \langle Z_i^{(k)} \rangle r^{+(k)}} \sum_i \langle Z_i^{(k)} \rangle \text{DNaseSum}_i^{+}. \qquad (41)
\end{aligned}
$$

Writing the above equation using $p^{+(k)}$ as defined in Equation 39, we get:

$$
\begin{aligned}
\frac{\partial L^{+}_{A}}{\partial r^{+(k)}} = &\sum_i \langle Z_i^{(k)} \rangle \psi\big(r^{+(k)} + \text{DNaseSum}_i^{+}\big) - \psi(r^{+(k)}) \sum_i \langle Z_i^{(k)} \rangle \\
&+ \left(\log \left(p^{+(k)} \right) + 1 - p^{+(k)} \right) \sum_i \langle Z_i^{(k)} \rangle - \frac{p^{+(k)}}{r^{+(k)}} \sum_i \langle Z_i^{(k)} \rangle \text{DNaseSum}_i^{+}. \qquad (42)
\end{aligned}
$$

Now the numerical optimization procedure referred to above is used to find the local maximum.

The Expectation-Maximization procedure was initialized by assigning the prior probabilities as described in Methods. The default initialization procedure can be overridden by directly providing

the initial values of the prior likelihoods. Our choice of the procedure was motivated by the fact that the total number of DNase-seq cuts in the vicinity is a very simple and reasonably accurate predictor for the motif instance to be bound. We expect that other procedures may perform comparably well, and to test this hypothesis we tried initializing the algorithm randomly, by putting $P(Z_i = 1)/P(Z_i = 0) = 100$ for a random 10% of motif instances. The random initialization performed surprisingly well in terms of AUC-PR and AUC-ROC when compared to the default one (Supplementary Figure 9). After applying the random initialization 5 times for each combination of TF, cell type and DNase I data source, we concluded that the random procedure missed the maximum found by the default procedure in 2.7% of the cases. Moreover, in no case the random procedure outperformed the default one, confirming the robustness of the latter.

We iterate the Expectation-Maximization procedure, in each iteration getting a revised vector of parameters Θ_t, until the posterior probabilities do not change by more than 0.001, i.e.

$$\max_{i,k} \left| P(Z_i = k \mid X_i, \Theta_{t+1}) - P(Z_i = k \mid X_i, \Theta_t) \right| < 0.001. \tag{43}$$

In most of the cases described here, the algorithm converged in less that 30 iterations.

4 Correlation between DNase I accessibility and motif score

The correlation was calculated for each TF motif, DNase-seq data source and cell type. We considered 500 bp long genomic windows, starting each 50 bp. For each window, we calculated the total number of DNase I reads mapped within the window, as a measure of DNase I accessibility. We also took the highest motif score for a genomic sequence within the window as the motif score for the whole window.

To allow for a balanced comparison between DNase I accessibility and motif score, we took all the windows overlapping the ChIP-seq peaks for the given TF, and additionally an equal number of randomly chosen windows not overlapping such a peak. Within these windows, we calculated the Spearman correlation coefficient between DNase I accessibility and motif score (Supplementary Figure 10). We found no clear trend between these correlation coefficients and Binding in Closed Chromatin (BCC) values (Supplementary Figure 10). This was also the case when we considered the correlation calculated within all the genomic windows, or only within the windows overlapping the ChIP-seq peaks.
